# Primitive haematopoiesis in the human placenta gives rise to macrophages with epigenetically silenced HLA-DR

**DOI:** 10.1038/s41467-023-37383-2

**Published:** 2023-03-30

**Authors:** Jake R. Thomas, Anna Appios, Emily F. Calderbank, Nagisa Yoshida, Xiaohui Zhao, Russell S. Hamilton, Ashley Moffett, Andrew Sharkey, Elisa Laurenti, Courtney W. Hanna, Naomi McGovern

**Affiliations:** 1 Centre for Trophoblast Research, University of Cambridge, Cambridge, UK; 2 Department of Pathology, University of Cambridge, Cambridge, UK; 3 Department of Haematology and Wellcome and MRC Cambridge Stem Cell Institute, University of Cambridge, Cambridge, UK; 4 Department of Physiology, Development and Neuroscience, University of Cambridge, Cambridge, UK; 5 Present Address: Life & Medical Sciences (LIMES) Institute, University of Bonn, Bonn, Germany

**Keywords:** Haematopoiesis, Monocytes and macrophages, Myelopoiesis

## Abstract

The earliest macrophages are generated during embryonic development from erythro-myeloid progenitors (EMPs) via primitive haematopoiesis. Although this process is thought to be spatially restricted to the yolk sac in the mouse, in humans, it remains poorly understood. Human foetal placental macrophages, or Hofbauer cells (HBC), arise during the primitive haematopoietic wave ~18 days post conception and lack expression of human leukocyte antigen (HLA) class II. Here, we identify a population of placental erythro-myeloid progenitors (PEMPs) in the early human placenta that have conserved features of primitive yolk sac EMPs, including the lack of *HLF* expression. Using in vitro culture experiments we demonstrate that PEMP generate HBC-like cells lacking HLA-DR expression. We find the absence of HLA-DR in primitive macrophages is mediated via epigenetic silencing of class II transactivator, CIITA, the master regulator of HLA class II gene expression. These findings establish the human placenta as an additional site of primitive haematopoiesis.

## Abstract (web summary)

The generation of primitive macrophages remains a poorly understood process in humans. Here, the authors identify placental erythro-myeloid progenitors that give rise to foetal macrophages in the early human placenta and demonstrate that epigenetic silencing of the class II transactivator leads to downregulation of HLA-DR in these cells.

## Introduction

The human placenta is a major organ that regulates the health of both the mother and foetus during pregnancy. It is the site of transfer of oxygen and nutrients to the foetus and acts as an important barrier against pathogens, amongst other functions. The placenta starts to form 5 days after conception and continues to grow and develop throughout pregnancy, up to birth. Placental macrophages, termed Hofbauer cells (HBC), first appear at ~18 days post conception^1^. They rapidly proliferate and are highly abundant within first-trimester placental villi, the functional units of the placenta. Throughout pregnancy, HBC remain the only significant foetal-derived immune cell population of the human placenta. HBC are unique amongst all tissue-resident macrophages, as equivalent cells are not found in the murine placenta^2,3^. Hence, primary human tissue must be used to develop our understanding of HBC ontogeny and function, and, consequently, HBC precursors remain poorly described^4^.

During gestation, haematopoiesis occurs in successive waves in different anatomical sites. The first primitive haematopoietic wave generates macrophages from a population of ‘early’ erythro-myeloid progenitors (EMPs), independently of a monocyte intermediate. These first macrophages are commonly termed ‘primitive’ macrophages. Later in gestation, transient definitive ‘late’ EMPs and haematopoietic stem cells (HSCs) give rise to ‘definitive’ macrophages via a monocyte stage^5–10^. Murine fate mapping models have established that the first embryonic macrophages are generated from EMPs in the yolk sac^10–12^, and it is widely accepted that the yolk sac is the sole site of primitive haematopoiesis^13^.

Previously, we observed that the transcriptome of HBC is very similar to that of yolk sac macrophages^2^. However, HBC are not likely to be generated in the yolk sac, as they have been identified in the placenta prior to the commencement of embryonic blood circulation^14^ and vascularisation of the placental mesenchyme^15^. Vitelline vessels of the yolk sac do not connect directly with the placenta, and foetal blood flow to the placenta via the umbilical cord is not established until ~10 post-conception weeks (PCW)^16,17^. Therefore, we sought to determine if HBC are generated de novo in the placenta. Previous work by others has identified CD34^+^CD43^+^ haematopoietic progenitors in pre-circulation human placental villi^15^. However, the identity of these progenitors and the cells they generate are poorly defined.

In this study, we characterise CD34^+^CD43^+^ progenitors in human placental villi. We find that these placental CD34^+^CD43^+^ progenitors have conserved features with yolk sac erythro-myeloid progenitors (EMP), including a lack of *HLF* expression, a defining feature of EMP. Hence, we termed placental CD34^+^CD43^+^ progenitors, as placental EMP (PEMP). We subsequently show that PEMP differentiate into primitive HLA-DR^neg^ HBC-like macrophages in vitro. We demonstrate that a conserved feature of human primitive macrophages across embryonic tissues, as well as extra-embryonic tissues, is a complete lack of expression of HLA class II molecules.

Analysis of the DNA methylation status of the class II transactivator (CIITA), indicates that the *CIITA* promoter is epigenetically silenced in HBC during the first trimester of pregnancy. However, as pregnancy progresses, we find that HBC begin to express HLA-DR. We show that first-trimester HLA-DR^neg^ HBC and third-trimester HLA-DR^pos^ HBC demonstrate highly similar DNA methylation patterns at the CIITA promoter and contrast starkly with HLA-DR^pos^ foetal blood monocytes that are derived from definitive HSCs. These findings suggest that primitive haematopoiesis in the placenta is the sole source of HBC throughout pregnancy. That is, foetal blood monocytes are unlikely to make a major contribution to the HBC population. Our data suggest that there is an erosion of epigenetic silencing throughout gestation, underpinning the observed transition of HBC from an HLA-DR^neg^ to HLA-DR^pos^ state.

These findings provide significant additional insight into the nature of human primitive macrophages, their epigenetic regulation of HLA-DR expression, and establish the human placenta as a site of primitive macrophage generation.

## Results

### Placental erythro-myeloid progenitors are present in early gestation

**Fig. 1:**
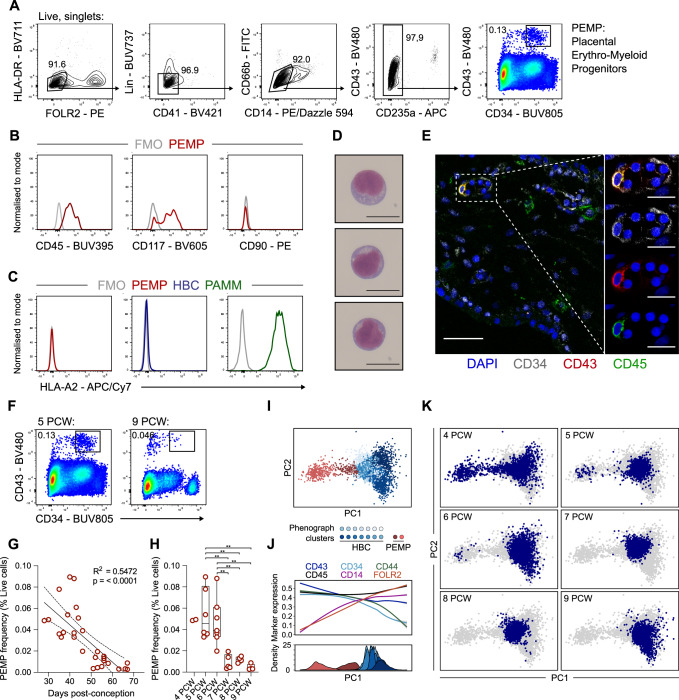
Placental erythro-myeloid progenitors (PEMP) are exclusively found early in gestation. **A** Representative flow cytometry gating strategy identifying CD43^+^CD34^+^ placental erythro-myeloid progenitors (PEMP) in a 5 post-conception week (PCW) sample. **B** Flow cytometric expression of CD45, CD117 (c-Kit) and CD90 in PEMP, data are representative of *n* = 2 donors for CD90 expression and *n* = 3 donors for CD45 and CD117 expression. FMO fluorescence minus one. **C** Relative flow cytometry expression of HLA-A2 in PEMP, Hofbauer cells (HBC) and placenta-associated maternal macrophages (PAMM) from a sample with an HLA-A2 allotype mismatch between mother and foetus. **D** Representative Giemsa-stained cytospins of PEMP isolated by FACS. Scale bars, 20 μm. Representative images of 3 independent experiments from *n* = 3 donors. **E** Identification of CD34^+^ (grey) CD43^+^ (red) CD45^+^ (green) PEMP within the villous stroma of a 5 PCW placental sample. Scale bars, main panel 100 μm, inset panels 20 μm. Representative images of 3 independent experiments from *n* = 1 donor. **F** Flow cytometric analysis of PEMP abundance in a 5 PCW and a 9 PCW sample. **G** Quantification of PEMP frequency via flow cytometry as a proportion of all live cells, plotted against post-conception age in days, or (**H**) grouped by week. *P* value and R^2^ calculated via Pearson’s Correlation. Boxplot centre lines represent the median, with box limits showing the upper and lower quartiles, and whiskers denoting minimum and maximum values (*n* = 28 donors). **I** Principle component analysis embedding of flow cytometry data of HBC, PEMP and any phenotypic intermediates from 12 placental samples (gating strategy for analysis detailed in Fig. S1F–I). Cells were subjected to Phenograph clustering and clusters are coloured according to their cellular identities. *n* = 12 donors, 2 × 4 PCW, 2 × 5 PCW, 2 × 6 PCW, 2 × 7 PCW, 2 × 8 PCW and 2 × 9 PCW. PC principal component. **J** Mean expression of key flow cytometric markers (top panel), and distributions of phenograph clusters (bottom panel) along PC1. **K** PCA embeddings as in (**I**), with cells from each timepoint highlighted in blue within each panel.

The observation that HBC are present 18 days post conception^14^, before foetal blood flow to the placenta begins, suggests that they arise from local progenitors. Indeed, although CD43^+^CD34^+^ progenitors have been described in the first-trimester placenta, they are poorly defined^15,18,19^. To investigate placental CD43^+^CD34^+^ progenitors, we designed a flow cytometry panel to analyse cell isolates of first-trimester placental villi. The panel was designed to exclude lymphocytes, erythrocytes, monocyte/macrophages and granulocytes, allowing the selection of CD43^+^CD34^+^ cells (Fig. 1A). Due to the early appearance of these cells, we hypothesize that the HLA-DR^−^FOLR2^−^CD41^−^Lin^−^CD66b^−^CD14^−^CD235a^−^CD34^+^CD43^+^ cells we have identified are PEMPs. PEMP express haematopoietic markers CD45 and c-KIT (CD117), but lack expression of CD90 (expressed by definitive HSCs in the bone marrow^20^ and placental fibroblasts^21^) (Fig. 1B), as well as EGFR and HLA-G (trophoblast markers)^22^ (Supplementary Fig. 1A.). Their foetal origin is confirmed by staining with HLA-allotype antibodies in comparison to foetal HBC and placenta-associated maternal macrophages (PAMM)^2^ (Fig. 1C, Supplementary Fig. 2A, B). Typical haematopoietic progenitor morphology is seen in Geimsa-stained cytospins of FACS-sorted PEMP (Fig. 1D, Supplementary Fig. 1B). Although rare, CD43^+^CD34^+^CD45^+^ PEMP are reliably observed within the stroma of first-trimester placental villi by immunofluorescence. We find PEMP in close association with CD34^+^ placental endothelium (Fig. 1E, Supplementary Fig. 1C), in line with previous reports of EMPs arising from endothelial cells^23^. Applying our flow cytometric gating strategy to placental samples of different gestational ages reveals that PEMP decrease significantly after the first weeks of pregnancy, and they are virtually absent from 7 PCW (Fig. 1F–H). The ratio of HBC:PEMP increases over gestation, consistent with PEMP initially seeding a minor population of macrophages which then proliferate and expand in situ (Supplementary Fig. 1D, E).

UMAP visualisation of flow cytometric data from a 4 PCW sample reveals some PEMP-HBC intermediates could be missed by our gating strategy (Supplementary Fig. 1F). To further investigate this population, we used Hyperfinder to derive a gating strategy to isolate HBC, PEMP and any potential phenotypic intermediates (Supplementary Fig. 1G–I). We applied this gating strategy to 2 donors from each PCW group, generated a principal component analysis (PCA) embedding and performed Phenograph clustering on the combined dataset (Fig. 1I, Supplementary Fig. 1J). Proposed PEMP-HBC differentiation is captured along the first principal component (PC1), and analysis of marker expression reveals one cluster of PEMP with elevated expression levels of CD14 and FOLR2 indicating that these cells are in transition from PEMP to HBC (Fig. 1J, Supplementary Fig. 1I). The intermediate cells are only seen at 4 PCW, and PEMP are observed up to 6 PCW, this suggests that PEMP-HBC differentiation only occurs at earlier timepoints. (Fig. 1K). Due to the scarcity of the intermediate cells and the nature of their phenotype, we were unable to capture these cells in our scRNAseq experiments. These findings demonstrate that the human placenta contains a transitory population of foetal haematopoietic progenitors, PEMP, which are found only at early timepoints in gestation.

### Transcriptomic and phenotypic heterogeneity of PEMP

**Fig. 2:**
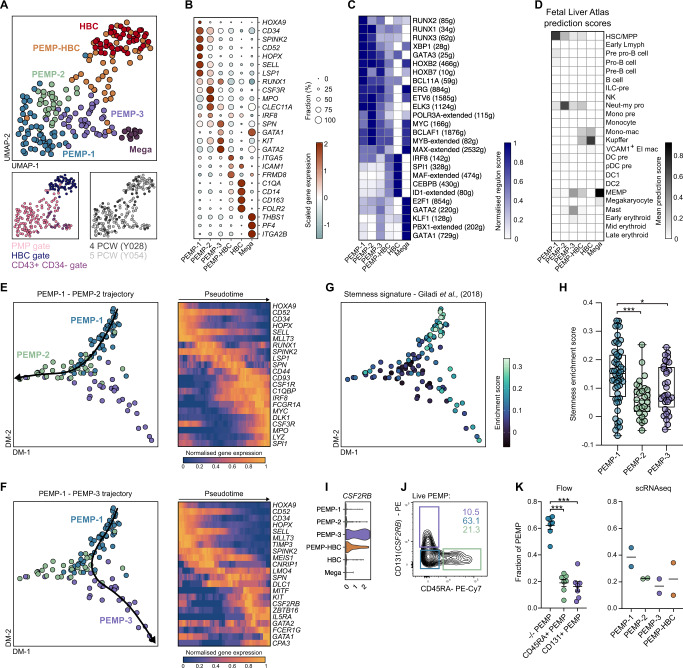
PEMP display transcriptomic and phenotypic diversity. **A** UMAP visualisation of 210 single-cell transcriptomes from 2 early placental samples. Cells are coloured by cluster identity. Bottom panels, cells are coloured by gate used for FACS isolation (left) and by donor (right). Mega: Megakaryocyte progenitors. **B** Dotplot heatmap displaying scaled log-normalised gene expression of key marker genes across the observed clusters. Dot size represents fraction of cells with nonzero expression. **C** Heatmap of selected inferred transcription factor activities (regulon scores) across the observed clusters, calculated via SCENIC analysis^30^. The number of genes associated with each regulon is listed in parentheses. **D** Heatmap showing mean prediction scores (transcriptomic similarity) of single cells within observed placental clusters using a human foetal liver atlas dataset as a reference^28^. Scores were calculated using the FindTransferAnchors function in Seurat (see Methods). MEMP (Megakaryocyte-Erythroid-Mast cell progenitors). **E** Left, Diffusion map embedding of PEMP scRNAseq data with PEMP-1 – PEMP-2 slingshot trajectory overlain (Black arrow). Right, Heatmap of smoothed normalized gene expression of differentially expressed genes across PEMP-1 – PEMP-2 slingshot trajectory. **F** Left, Diffusion map embedding of PEMP scRNAseq data with PEMP-1 – PEMP-3 slingshot trajectory overlain (Black arrow). Right, Heatmap of smoothed normalized gene expression of differentially expressed genes across PEMP-1 – PEMP-3 slingshot trajectory. **G** Diffusion map embedding of PEMP scRNAseq data with heatmap overlay of haematopoietic stem cells stemness gene signature enrichment scores. **H** Quantification of single-cell enrichment scores across PEMP subsets. *P* values calculated by two-tailed Mann–Whitney test. *p ≤ 0.05 (exact *p* value = 0.0457), ***p ≤ 0.001 (exact *p* value = 0.0001). Boxplot centre lines represent the median, with box limits showing the upper and lower quartiles, and whiskers denoting minimum and maximum values. Cells from *n* = 2 biologically independent placental samples. **I** Violin plot of *CSF2RB* log-normalised gene expression across observed clusters. **J** Analysis of CD131 and CD45RA expression within PEMP via flow cytometry revealing three populations: CD45RA^−^CD131^−^ PEMP (blue), CD45RA^+^ PEMP (green) and CD131^+^ PEMP (purple). **K** Quantification of PEMP populations via flow cytometry (*n* = 4 donors) (left panel) and scRNAseq (*n* = 2 donors) clusters within the PEMP gate (right panel). *P* values calculated by two-tailed Mann–Whitney test. Flow cytometry quantification data are presented as mean ± SEM, scRNAseq quantification data are presented as mean alone.

To confirm that the cells we identified as PEMP are true haematopoietic progenitors we first looked for haematopoietic progenitors in a scRNAseq atlas of the first trimester-placenta^24^. Integration of this dataset with other foetal scRNAseq datasets (CS7 embryo^25^, CS10 embryo^26^, CS11 YS^27^, CS15 aorta-gonad mesonephros (AGM)^26^, foetal liver^28^ and foetal bone marrow^29^) reveals very few placental cells in the combined haematopoietic progenitor cell cluster (Supplementary Fig. 3A). This is likely due to the scarcity of PEMP, and their presence only early in gestation. Therefore, we performed SmartSeq2 scRNAseq on FACS purified CD43^+^CD34^+^ PEMP, HBC, an undefined population of CD43^+^CD34^−^ cells, and endothelial cells from a 4 PCW and a 5 PCW sample (Supplementary Fig. S2A). After pre-processing, and the removal of 35 endothelial cells (*CDH5*^+^, *KDR*^+^) and 10 erythrocytes (*HBZ*^+^, *HBE1*^+^) from the dataset, 210 cells were annotated into 6 clusters based on their gene expression (Fig. 2A). All cells profiled are foetal in origin, and each cluster contains cells derived from each donor (Supplementary Fig. 3B, C).Three populations of PEMP can be identified (PEMP-1,2,3), as well as HBC, megakaryocyte progenitors (Mega), and a population composed of both PEMP and HBC (PEMP-HBC). Cells within the three PEMP clusters are primarily derived from the PEMP gate during FACS isolation and are proliferating (Supplementary Fig. 3D–F). Analysis of differentially expressed genes (DEGs) between the clusters reveals heterogeneity within the PEMP compartment, including cell surface markers and transcription factors (Supplementary Fig. 4A–D). PEMP-1 express typical haematopoietic progenitor genes including *HOXA9*, *CD34*, *SPINK2*, *HOPX* (Fig. 2B). PEMP-2 and PEMP-3 are myeloid and megakaryocyte/erythrocyte/mast cell biased progenitors respectively, based on their expression of known lineage-specific genes *IRF8*, *MPO*, *CLEC11A, CSF3R*, *GATA1*, *GATA2* and *KIT* (Fig. 2B). HBC and megakaryocyte progenitor clusters express known macrophage (*C1QA*, *CD14*, *CD163*) and megakaryocyte (*THBS1*, *PF4*, *ITGA2B*) genes (Fig. 2B).

To further probe PEMP identity we leveraged SCENIC^30^ to infer transcription factor (regulon) activities within each cluster (Supplementary Fig. 4E, F). This indicates that PEMP are under the control of transcription factors important in haematopoietic progenitor identity and fate, including RUNX1, RUNX3, GATA3 and ERG (Fig. 2C). In line with our gene expression data, PEMP-2 and PEMP-3 express transcription factors typical of myeloid lineages (MYC, MYB, IRF8) and megakaryocyte/erythroid/mast cell lineages (GATA1, GATA2, KLF1, PBX1) respectively (Fig. 2C, Supplementary Fig. 4G).

Using the *FindTransferAnchors* function in Seurat we analysed the transcriptomic similarities between our clusters and annotated clusters from a recently published dataset of human foetal liver haematopoiesis^28^. Each PEMP cluster displays similarity to distinct foetal liver progenitors; PEMP-1 to HSC/MPP, PEMP-2 to neutrophil-myeloid progenitors and PEMP-3 to megakaryocyte-erythroid-mast cell progenitors (Fig. 2D, Supplementary Fig. 5A). A minor population of cells within the PEMP-3 cluster display high transcriptomic similarity to mast cells (Fig. 2D, Supplementary Fig. 5B). These cells express canonical mast cell genes (*HDC*, *MITF*, *CPA3*, *KIT*), but lack the expression of *FCER1A* mRNA (Supplementary Fig. 5C). Flow cytometry analysis confirms the presence of foetal CD200R^+^CD117^+^FCεR1^−^ mast cells in early placental samples (Supplementary Fig. 5D). Placental mast cell FCεR1 expression is consistent with previous reports describing foetal mast cells^31,32^ but further investigation of these is beyond the scope of this study.

These analyses suggest that PEMP-1 can differentiate along two distinct pathways governed by different transcriptional programmes: either towards the myeloid lineage (PEMP-2) or towards the megakaryocyte-erythroid-mast cell lineages (PEMP-3). To investigate this further we performed slingshot analysis^33^ on PEMP subsets, revealing two branching trajectories originating from PEMP-1, towards either PEMP-2 or PEMP-3 (Fig. 2E, F). PEMP-1 are highly enriched for a gene signature associated with haematopoietic stem cell quiescence and “stem-ness”^34^, supporting their proposed position at the root of these differentiation trajectories (Fig. 2G,H). We next aimed to identify cells of each PEMP lineage at the protein level. *CSF2RB* (the gene encoding CD131) is expressed specifically in PEMP-3 (Fig. 2I, Supplementary Fig. 4B), and CD45RA is known to be expressed by definitive myeloid-biased progenitors within the adult bone marrow^35^. Using these two markers we can thus identify three PEMP populations via flow cytometry, representing our three scRNAseq data clusters (CD45RA^−^CD131^−^ = PEMP-1, CD45RA^+^ = PEMP-2, CD131^+^ = PEMP-3) (Fig. 2J, Supplementary Fig. 6A, B). To further probe PEMP phenotypic heterogeneity, we examined their expression of CD52, CD200 and CD64 (*FCGR1A*), markers which were found to be differentially expressed across PEMP subsets in our scRNAseq data (Supplementary Fig. 6C). We find that CD52, CD200 and CD64 protein expression patterns within CD45RA^-^CD131^-^ PEMP-1, CD45RA^+^ PEMP-2 and CD131^+^ PEMP-3 match mRNA expression observed across PEMP scRNAseq clusters (Supplementary Fig. 6D, E), suggesting that CD45RA and CD131 expression reliably identify PEMP-1, PEMP-2, and PEMP-3. We additionally find that phenotypic subsets of PEMP are similarly abundant to analogous scRNAseq clusters (Fig. 2K), validating our observed transcriptional heterogeneity. In summary, our findings confirm that PEMP are true haematopoietic progenitors, and suggest that PEMP seed diverse haematopoietic populations within the placenta.

### PEMP do not express *HLF*

**Fig. 3:**
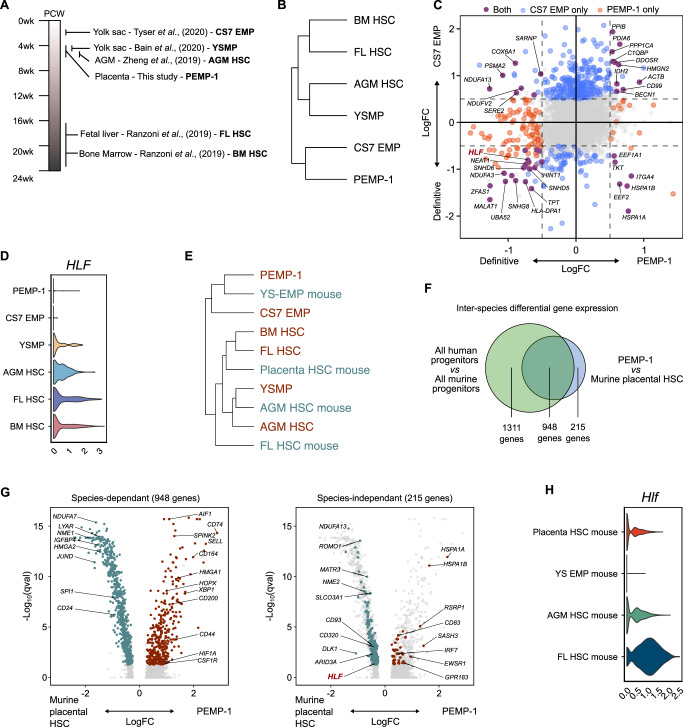
PEMP display characteristics of primitive erythro-myeloid progenitors. **A** Schematic representation of human haematopoietic progenitor populations used for comparative analysis^25–27,29^. CS Carnegie Stage, EMP Erythro-myeloid progenitors, YSMP Yolk sac myeloid-biased progenitors, AGM Aorta-gonad mesonephros, FL Foetal liver, BM Bone marrow, HSC Haematopoietic stem cell. **B** Hierarchical clustering dendrogram depicting transcriptomic similarity between human foetal progenitor populations, calculated using gene expression distance matrix from the integrated dataset (Fig. S7A). **C** Scatter plot showing differentially expressed genes (DEGs) between PEMP-1 and definitive progenitors (YSMP, AGM HSC, FL HSC and BM HSC) on the x-axis and CS7 EMP and definitive progenitors on the y-axis. Genes significantly differentially expressed (adjusted *p* value <0.05) in both comparisons, PEMP-1 alone and CS7 EMP alone are shown in purple, orange and blue respectively. Selected genes which were differentially expressed in both CS7 EMP and PEMP-1 are highlighted. **D** Violin plot of *HLF* log-normalised gene expression across human foetal haematopoietic progenitors. **E** Hierarchical clustering dendrogram depicting transcriptomic similarity between human and murine^37,38^ foetal progenitor populations, calculated using gene expression distance matrix from the integrated dataset (Fig. S7C). YS – Yolk sac. **F** Venn diagram depicting the overlap of DEGs calculated between all human and all murine foetal haematopoietic progenitors, and DEGs calculated between PEMP-1 and murine placental HSC alone. **G** Volcano plots showing DEGs between PEMP-1 and murine placental HSC. Left panel: genes which overlap with DEGs calculated between all human and all murine foetal haematopoietic progenitors. Right panel: genes which do not overlap. **H** Violin plot of *Hlf* log-normalised gene expression across murine foetal haematopoietic progenitors.

We predict that PEMP are primitive progenitors because of their temporal distributions throughout gestation, and because the progeny of definitive HSCs such as monocytes are not found within first-trimester placental villi^2^. We therefore compared their transcriptomes with other populations of human foetal haematopoietic progenitors (Fig. 3A, Supplementary Fig. 7A, B)^25–27,29^. Hierarchical clustering of the integrated dataset reveals a broad division between the progenitor populations, with PEMP-1 clustering most closely to primitive CS7 EMPs, away from yolk sac myeloid-biased progenitors (YSMPs), aorta gonad mesonephros (AGM), foetal liver and bone marrow HSCs (Fig. 3B). The clustering of YSMP with HSCs suggests that these cells are definitive progenitors. To determine conserved features of primitive EMP we calculated differentially expressed genes between both CS7 EMPs and PEMP-1 and definitive progenitors (Fig. 3C). *HLF* is not expressed in either CS7 EMPs or PEMP, but is present in all other lineages, including YSMP (Fig. 3D). *HLF* is known to be constitutively expressed in murine definitive HSC, but not EMPs^36^.

Recently, the haematopoietic progenitor compartment in the murine placenta was characterised in detail^37^. To compare murine placental HSC with human PEMP we performed an inter-species transcriptional analysis (Supplementary Fig. 7C, D). Hierarchical clustering of the integrated inter-species dataset reveals that murine placental HSC do not cluster with human primitive CS7 EMP or PEMP-1, but are instead transcriptionally similar to definitive HSCs^38^ (Fig. 3E, Supplementary Fig. 7D). We find 1,163 DEGs (adjusted *p*-value <0.05) between PEMP-1 and murine placental HSC (Fig. 3F). 948 (81.5%) are dependent on differences between species alone and are seen in other comparisons of human-murine progenitors. 215 DEGs (18.5%) are independent of differences between species comparisons (Fig. 3F, G) and include *HLF*. Unlike PEMP-1, there is comparable *Hlf* expression between murine placental HSC and definitive AGM HSC (Fig. 3H). Together these data suggest that PEMP are primitive EMPs, and that the murine placental HSC that have been identified thus far are not analogous to PEMP, as they have transcriptional features of definitive progenitors.

### PEMP generate primitive HLA-DR^neg^ HBC-like cells in vitro

**Fig. 4:**
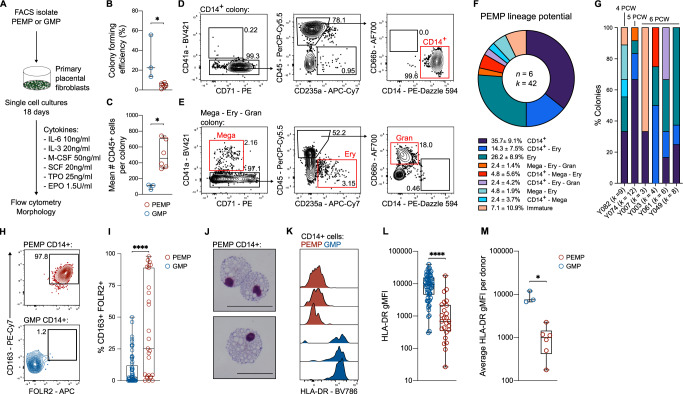
PEMP generate HLA-DR^neg^ HBC-like cells in vitro. **A** Schematic depicting experimental design for PEMP single-cell culture assay. **B** Boxplot depicting total colony-forming efficiency of adult peripheral blood GMP (Granulo-myeloid progenitors) and PEMP. GMP *n* = 3 donors, PEMP *n* = 6 donors. *P*-value calculated by two-tailed Mann–Whitney test. Exact *p* value = 0.0238. **C** Boxplot depicting mean number of CD45^+^ cells per colony per donor. GMP *n* = 3 donors, PEMP *n* = 6 donors. *P* value calculated by two-tailed Mann–Whitney test. Exact *p* value = 0.0238. **D** Representative flow cytometry analysis of a single-cell PEMP-derived colony which yielded CD14^+^ cells. **E** Representative flow cytometry analysis of a single-cell PEMP-derived colony which yielded megakaryocytes (Mega), erythroid cells (Ery) and granulocytes (Gran). **F** Donut plot showing lineage potential of PEMP defined by single-cell culture and flow cytometry analysis. ‘Immature’ denotes CD45^+^ colonies which did not contain any other defined immune cell lineages. (*n* = 6 donors, *k* = 42 colonies generated). **G** Stacked bar charts showing lineage potential of PEMP for each donor profiled. PCW age of the donors are added as labels above the bar chart. **H** Analysis of CD163 and FOLR2 expression in CD14^+^ colony derived from single PEMP and GMPs via flow cytometry, and **I** boxplot quantification of co-expression for all CD14^+^ colonies profiled (GMP *n* = 3 donors, *k* = 61 colonies) (PEMP *n* = 6 donors, *k* = 25 colonies). *P* value calculated by two-tailed Mann–Whitney test. *P* value <0.0001. **J** Representative Giemsa-stained cytospins of cells from CD14^+^ colonies derived from a single PEMP. Scale bars, 50 μm. Representative images from *n* = 4 donors. **K** Analysis of HLA-DR expression in CD14^+^ colony derived from single PEMPs and GMPs via flow cytometry. Each histogram represents a colony derived from an independent donor. **L** Boxplot quantification of HLA-DR expression in CD14^+^ colony derived from single PEMPs and GMPs via flow cytometry for all colonies profiled (GMP *n* = 3 donors, *k* = 61 colonies) (PEMP *n* = 6 donors, *k* = 25 colonies). *P* value calculated by two-tailed Mann–Whitney test. Exact *p* value = <0.0001. **M** Boxplot depicting median HLA-DR expression of all CD14^+^ colonies per donor. (GMP *n* = 3 donors) (PEMP *n* = 6 donors). All boxplot centre lines represent the median, with box limits showing the upper and lower quartiles, and whiskers denoting minimum and maximum values. Exact *p* value = 0.0238. *P ≤ 0.05, **P ≤ 0.01; ***P ≤ 0.001, ****P ≤ 0.0001.

We have previously shown that a defining characteristic of first-trimester HBC and yolk sac macrophages is a lack of HLA-DR expression^2^ (see also Supplementary Fig. 9A, B). As proof-of-concept, we next explored whether PEMP can differentiate into HLA-DR^neg^ primitive macrophages. To mimic the placental microenvironment, PEMP were cultured for 18 days as single cells on monolayers of primary first-trimester placental fibroblasts, supplemented with cytokines that promote macrophage differentiation (Fig. 4A, Supplementary Fig. 8A,). As a control for definitive myeloid cell differentiation, we performed identical experiments using granulo-myeloid progenitors (GMPs) isolated from adult peripheral blood (Fig. 4A, Supplementary Fig. 2C). The colony-forming efficiency of PEMP is significantly lower than definitive GMPs (Fig. 4B). Despite this, PEMP generated larger colonies than GMP (Fig. 4C, Supplementary Fig. 8I), indicating they are more proliferative. PEMP single-cell cultures give rise to morphologically and phenotypically distinct colonies (Fig. 4D, E, Supplementary Fig. 8B–F). They generate diverse haematopoietic lineages, including CD14^+^ cells, megakaryocytes, erythroid cells, and in rare cases granulocytes (Fig. 4D–F, Supplementary Fig. 8F), but display limited multipotency (Fig. 4F, G). This is in contrast to YSMP, that are poor at generating erythrocytes^27^. Most colonies contain CD14^+^ cells (~60%), and these were generated in all donors (Fig. 4G). PEMP that give rise to CD14^+^ and megakaryocyte colonies in single-cell cultures display increased expression of CD45RA and CD131 respectively (Supplementary Fig. 8G, H), suggesting that transcriptionally diverse populations of PEMP (Fig. 2) have distinct fates.

To determine if PEMP-derived CD14^+^ cells are macrophages, we analysed their expression of CD163 and FOLR2, two surface receptors highly expressed by HBC^2^. PEMP seem more disposed to differentiate into CD163^+^ FOLR2^+^ macrophages (Fig. 4H) than GMP (Fig. 4I, Supplementary Fig. 8J). PEMP-derived CD14^+^ cells are large and highly vacuolated, typical of HBC (Fig. 4J). Critically, PEMP-derived CD14^+^ cells do not express HLA-DR, while GMP-derived CD14^+^ cells do (Fig. 4K-M, Supplementary Fig. 8K). In conclusion, our findings suggest PEMP found in early pregnancy produce HBC.

### A lack of HLA Class II expression is a conserved feature of primitive macrophages

**Fig. 5:**
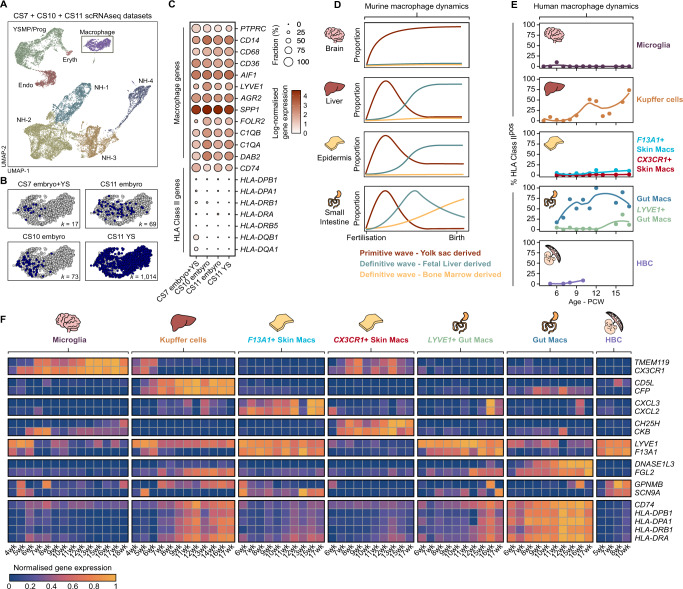
Human primitive macrophages are HLA-DR^neg^. **A** UMAP visualisation of 18,655 human early embryo single-cell transcriptomes from^25–27^, NH Non-haematopoietic, Endo – Endothelial, YSMP Yolk sac myeloid-biased progenitors, Prog – Haematopoietic progenitors, Eryth – Erythrocytes. **B** UMAP visualisation of early embryonic macrophage single-cell transcriptomes, cells from each study are highlighted in blue within each panel. Number of cells from each dataset (*k*) are annotated on each panel. CS Carnegie Stage, YS Yolk sac. **C** Dotplot heatmap displaying log-normalised gene expression of genes associated with macrophage identity and HLA Class II expression, within early human macrophages. Dot size represents fraction of cells with nonzero expression. **D** Diagram depicting the previously established contributions of primitive and definitive haematopoiesis to tissue-resident macrophage populations across murine organs. Redrawn and modified from^13^. **E** Line graphs showing the temporal dynamics of HLA Class II expression in macrophages from different human foetal tissues^24,27,28,39–41^. Cells are considered HLA Class II^pos^ if they display non-zero expression of *HLA-DRA*, *HLA-DRB1*, *HLA-DPA1*, *HLA-DPB1*, *HLA-DQA1* and *HLA-DQB1*. HBC Hofbauer cells, PCW Post-conception weeks. **F** Normalised gene expression heatmap of marker genes and HLA Class II genes in human foetal macrophages across developmental time.

PEMP generate HLA-DR^neg^ macrophages while GMP generate HLA-DR^pos^ macrophages under the same culture conditions. This suggests a lack of HLA-DR expression is determined by the ontogeny of HBC rather than their local microenvironment. Furthermore, a conserved feature of EMP across two distinct anatomical sites, the yolk sac and placenta, is the generation of macrophages that completely lack expression of HLA class II molecules (Supplementary Fig. 9A, B)^2^. Hence, we sought to determine if a lack of HLA-DR expression can be used to identify ‘primitive macrophages’ across other embryonic tissues. To address this, we analysed publicly available scRNAseq gene expression data from a range of early human embryonic samples - Carnegie stage 7 to 11 (CS7 to CS11, equivalent to approximately 19-26 days post conception)^25–27^. Combined analysis reveals a population of macrophages conserved across all datasets (Fig. 5A, B, Supplementary Fig. 9C, D) that express prototypical macrophage genes including *PTPRC*, *CD68*, *CD14*, *AGR2*, *CD36* and *AIF1* (Fig. 5C), and that uniformly lack the expression of all HLA class II genes (*HLA-DRA, HLA-DRB1, HLA-DRB5, HLA-DQA1, HLA-DQB1*) (Fig. 5C).

Fate-mapping models by others have established macrophage turnover in murine tissues during foetal development (Fig. 5D)^5–7,12,13^. We propose that the dynamics of gene expression in foetal macrophages, in particular HLA class II expression, can be used to visualise how primitive macrophages are replaced by definitive macrophages across human foetal tissues (Fig. 5E,F, Supplementary Fig. 10A-D)^24,27,28,39–41^. We find macrophages adopt tissue-specific gene signatures in a time-dependent fashion, for example *TMEM119* and *CX3CR1* in microglia and *CD5L* and *CFP* in Kupffer cells (Fig. 5F). The temporal expression dynamics of HLA class II genes in human foetal macrophages map closely to ontogenies of murine macrophages established by fate-mapping models (Fig. 5D–F)^13^. For example, microglia, which have only a minimal contribution from definitive haematopoiesis^5,12^, remain negative for HLA class II up to 18 PCW (Fig. 5E, F, Supplementary Fig. 10D). In contrast, Kupffer cells, where primitive cells are replaced by foetal liver-derived definitive cells in mice^5^, are HLA class II-negative at early timepoints (4–7 PCW) but positive later (11wk-17wk PCW) (Fig. 5E,F, Supplementary Fig. 10D). Therefore, a conserved feature of early human primitive macrophages in embryonic tissues (including placental HBC) is the lack of expression of HLA class II genes.

### CIITA is epigenetically silenced during primitive haematopoiesis

**Fig. 6:**
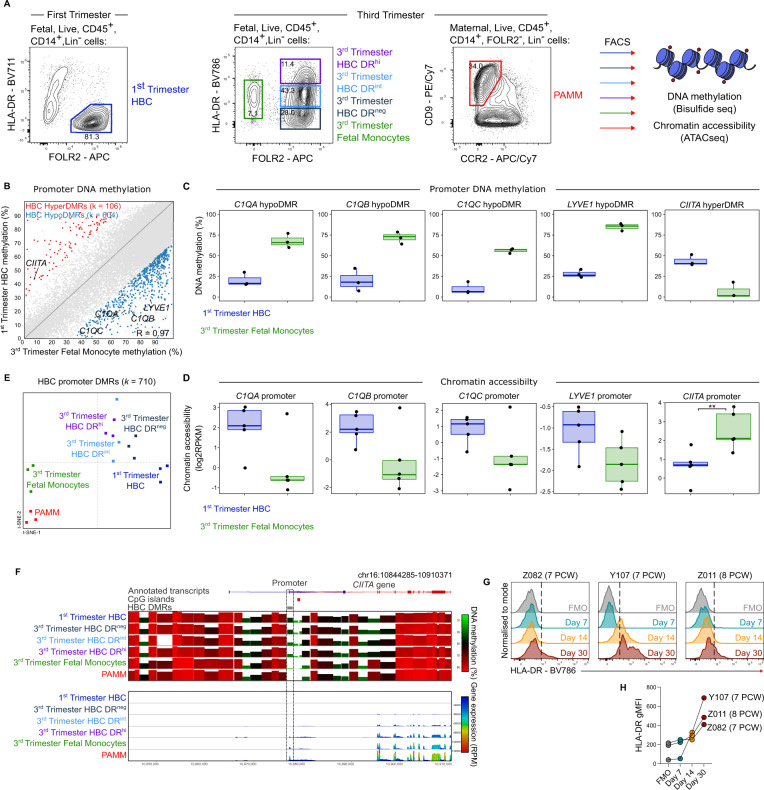
*CIITA* is epigenetically silenced during primitive haematopoiesis. **A** Flow cytometric data illustrating the gating strategy used to select HBC from first-trimester placenta (left plot), and foetal blood monocytes (HLA-DR^pos^FOLR2^neg^ cells, green gate), HBC subsets FOLR2^pos^HLA-DR^neg^ (black gate), HLA-DR^int^ (blue gate) HLA-DR^hi^ (purple gate) (left plot), and PAMM (CD9^+^CCR2^neg^ cells) (red gate) from third-trimester placentas. Right panel indicates experimental design. **B** Scatterplot showing average DNA methylation for 50 CpG windows overlapping gene promoters (*k* = 52,619 promoters) for first-trimester Hofbauer cells (HBCs) (*n* = 3 donors) compared to term foetal monocytes (*n* = 3 donors). Differentially methylated regions (DMRs) that were hyper- (*k* = 106) and hypo-methylated (*k* = 604) in HBCs compared to foetal monocytes are shown in blue and red, respectively. **C** Boxplots showing levels of DNA methylation in first-trimester HBCs (*n* = 3 donors) compared to term foetal monocytes (*n* = 3 donors) for a selection of active (*C1QA*, *C1QC*, *C1QB*, and *LYVE1*) and repressed genes (*CIITA*) in first-trimester HBCs. The boxplot centre line is the median, with box limits showing the upper and lower quartiles, whiskers as 1.5x interquartile range and overlaid dots show each of the data points. A genome-wide logistic regression with Benjamini-Hochberg correction for multiple comparisons, yielded the following *p*-values: C1QA (*p* < 0.001), C1QC (*p* < 0.001), C1QB (*p* < 0.001), LYVE1 (*p* < 0.001), CIITA (*p* < 0.001). **D** Boxplots show levels of chromatin accessibility in first-trimester HBCs (*n* = 5 donors) compared to third-trimester foetal monocytes (*n* = 5 donors) for a selection of active (*C1QA*, *C1QC*, *C1QB*, and *LYVE1*) and repressed genes (*CIITA*) in first-trimester HBCs. The asterisks show significant pairwise comparisons (**p = 0.007, one-tailed *t*-test) after correction for multiple comparisons using Bonferroni correction. All other comparisons: C1QA (*p* = 0.0261), C1QC (*p* = 0.0546), C1QB (*p* = 0.0321), LYVE1 (*p* = 0.0321), did not reach significance after Bonferroni correction. The boxplot centre line is the median, with box limits showing the upper and lower quartiles, whiskers as 1.5x interquartile range and overlaid dots show each of the data points. **E** tSNE visualisation of DNA methylation data shows the replicates for first-trimester HBCs, term HLA-DR negative (DR^neg^), HLA-DR intermediate (DR^int^) and HLA-DR high (DR^hi^) HBCs, term foetal monocytes and maternal PAMM (Placenta-associated maternal macrophages) from first and third-trimester placentas. Dimensionality reduction and clustering was based on the DNA methylation values for the DMRs identified in first-trimester HBCs compared to foetal monocytes. **F** Plot shows average DNA methylation (top) and gene expression (bottom) for each cell type across the *CIITA* gene locus. DNA methylation is shown for using 50-CpG windows and gene expression is shown as RPM for 100 bp windows, using a 10 bp step. The *CIITA* promoter is highlighted in the dashed box. **G** Histograms illustrating HLA-DR cell surface expression profile of first-trimester HBC after culture in vitro for up to 30 days (*n* = 3 donors). Dotted line marks positive expression in comparison with the negative control. FMO fluorescence minus one. **H** Quantification of HLA-DR expression.

To understand how HLA-DR expression is regulated in primitive macrophages, we carried out bisulphite sequencing of HBC and foetal monocytes, which arise from primitive and definitive haematopoiesis, respectively. The term placenta is a highly vascularised organ and foetal blood monocytes can be readily isolated from term placental digests by FACS (Fig. 6A, Supplementary Fig. 2D,E). First-trimester HBC were purified by FACS as described previously^2^ illustrated in Fig. 6A, Supplementary Fig. 2A,B. We find that promoter DNA methylation patterns are distinct between HBC and foetal monocytes, with 710 promoters showing significant differential DNA methylation of >30% (Fig. 6B). The methylation of the *C1QA*, *C1QB*, *C1QC* and *LYVE-1* promoters in HBC is low (Fig. 6C), consistent with high expression of these transcripts in primitive macrophages (Fig. 5C, Supplementary Fig. 11A). The class II transactivator, *CIITA*, is the master regulator of HLA Class II expression^42^. Our data shows that the *CIITA* promoter is significantly more methylated in first-trimester HBCs than foetal monocytes (Fig. 6C, Supplementary Fig. 11A). To further support that differential DNA methylation of the *CIITA* promoter, as well as the other candidate differentially methylated regions (DMRs) we have identified, is linked to promoter accessibility as well as gene expression, we generated ATAC-sequencing libraries from first-trimester HBC (*N* = 5) and term foetal monocytes (*N* = 5). These analyses revealed that DMRs were strongly associated with differential chromatin accessibility (Fig. 6D, Supplementary Fig. 11B). We find DNA methylation at the *CIITA* promoter in first-trimester HBC was significantly associated with closed chromatin state compared to the unmethylated promoter in foetal monocytes (Fig. 6C, D). While this does not exclude the possibility of other epigenetic factors contributing to gene regulation, these data provide evidence that DNA methylation is functioning in a repressive manner in HBC and foetal monocytes to regulate HLA-DR expression.

Flow cytometric analysis reveals that by term, HBC display variable levels of HLA-DR expression (Fig. 6A). We used the 710 promoter DMRs defined in first-trimester HBC to delineate whether these populations arise from primitive or definitive haematopoiesis. Our results demonstrate term HLA-DR^neg^, HLA-DR^int^ and HLA-DR^hi^ HBC show highly similar DNA methylation patterns to first-trimester HLA-DR^neg^ HBC (Fig. 6E, F, Supplementary Fig. 11A, C).

As the *CTIIA* promoter is hypermethylated across all term HBC subsets in comparison with foetal blood monocytes (Fig. 6F, Supplementary Fig. 11A), it is unlikely that foetal blood monocytes make a major contribution to HBC found in the term placenta. These findings support our hypothesis that primitive haematopoiesis in the placenta is the sole source of HBC throughout pregnancy.

Furthermore, we find that promoter DNA methylation at *CIITA* is anti-correlated with gene expression across HBC and monocyte populations (Linear correlation coefficient = −0.92, *p* < 0.01) (Fig. 6F). This suggests that there is an erosion of epigenetic silencing throughout pregnancy, underpinning their transition from an HLA-DR^neg^ to HLA-DR^pos^ state.

Finally, using in vitro culture assays, we find that first-trimester HBC can acquire low levels of HLA-DR surface protein expression after 30 days in culture (Fig. 6G, H). However, their HLA-DR expression profile does not fully reflect that of term HBC, suggesting that longer culture periods or additional stimuli are required.

Together these data suggest that the lack of HLA Class II expression in primitive macrophages is mediated through hypermethylation of the *CIITA* promoter. Throughout pregnancy there appears to be an erosion of epigenetic silencing at this locus, permitting the gradual acquisition of HLA Class II expression in primitive macrophages over time.

## Discussion

The yolk sac is widely recognised as the sole site of primitive haematopoiesis, and the source of the first macrophages that seed embryonic tissues. These macrophages are crucial for development of embryonic organs; they aid in the remodelling of tissues via scavenger activities^2^, the enucleation of erythrocytes^15^, strongly support vasculogenesis^43^ and display numerous tissue-specific imprinted functions^44^. However, the placenta is not directly connected to the yolk sac and foetal blood flow to the placenta only becomes fully established at ~10 PCW. Yet, the first-trimester human placenta is densely packed with macrophages, the first being identified at this site at ~18 days post conception. Our work sought to determine the source of HBC, and indicates that the human placenta generates its own pool of macrophages, through primitive haematopoiesis.

There have been previous reports of CD34^+^ progenitors identified in first-trimester placentas^15,18,19,45^, but these cells have been poorly defined till now. Our findings further characterise these HBC progenitors. We now show that placental primitive haematopoiesis is not supplanted by a transient definitive wave, as in the yolk sac. Instead, PEMP maintain their ability to generate HLA-DR^neg^ primitive macrophages up until 6 PCW, when their numbers begin to fall. The temporal distribution of PEMP currently remains unexplained but it is possible that they have limited capacity for long-term self-renewal and so begin to disappear as pregnancy continues. Alternatively, an external cue might cause PEMP demise, and the likely candidate is the sharp increase in oxygen tension and oxidative stress which occurs within placental villi between 6–9 PCW when the haemochorial circulation is established^46^. Using in vitro assays, we demonstrate that PEMP give rise to HLA-DR^neg^ HBC-like cells. Several studies have shown that macrophage populations exhibit distinct transcriptional signatures that are imprinted by their host tissue^44,47^. Hence, we consider it unlikely that the HLA-DR^neg^ HBC-like cells generated from PEMP in vitro are bona fide HBC.

Transcriptomic comparisons within and between species confirmed that PEMP are true EMPs, and they lack the expression of *HLF*, a transcription factor constitutively expressed in definitive HSCs^36,48^ and a critical regulator of HSC quiescence^49^. Although it cannot be ruled out that PEMP traffic to the placenta instead of being generated in situ, this is unlikely as HBC are found in the placenta prior to the commencement of cardiac contractions at day 21 post conception^50^ and the placental vasculature does not mature until ~10 PCW^16,17^. Furthermore, cells such as HLA-DR^pos^ monocytes, from the transient definitive wave of haematopoiesis that occurs in the yolk sac, are not present at equivalent time points in the placenta^2^. We also find PEMP in close association with developing placental endothelium. Finally, PEMP are transcriptionally and functionally distinct from YS progenitors (YSMP) found at equivalent developmental ages because the latter express *HLF* and are poor at generating erythrocytes^27^. These findings suggest that HBC are generated de novo in the human placenta.

Our work also suggests that lack of all isoforms of the HLA class II genes is an intrinsic property of human primitive macrophages, regulated by hypermethylation and chromatin inaccessibility of the *CIITA* promoter. These data are important as they allow us to distinguish primitive from definitive human macrophages early in gestation. We use this finding to show that the dynamics of macrophage turnover in human foetal tissues correlates well with dynamics found in the mouse^5–7,12,13^. However, we cannot exclude the possibility that these data may reflect primitive macrophage acquisition of HLA-DR expression at differential rates across tissues, instead of reflecting macrophage turnover.

Indeed, there is a temporal increase in HLA class II expression by primitive macrophages, such as, microglia^51,52^ and HBC. By term, three HBC subsets can be identified, based on their HLA-DR expression profile, HLA-DR^neg^, HLA-DR^int^ and HLA-DR^hi^ HBC. Our results demonstrate that HLA-DR^neg^, DR^int^ and DR^hi^ HBC show highly similar DNA methylation patterns to first-trimester HLA-DR^neg^ HBCs and not foetal blood monocytes. The *CTIIA* promoter is hypermethylated in all term HBC subsets, in comparison with foetal blood monocytes. DMRs in tissue macrophages are significantly shaped by the microenvironment^44,53^, however, if any of the term HBC subsets were derived from foetal blood monocytes, we would expect their *CTIIA* promoter to be similarly hypomethylated. This suggests that foetal blood monocytes do not make a major contribution to any of the HBC populations found in term placenta. Instead, we propose that primitive haematopoiesis in the placenta is the sole source of HBC throughout pregnancy and the acquisition of HLA-DR by HBC is likely due to erosion of epigenetic silencing of the promoter of the master regulator CIITA. Beyond CIITA, it is likely that many of the DMRs are of biological importance and interest to study in future work.

Functionally, the acquisition of HLA-DR by foetal macrophages coincides with the generation of foetal T cells^54^, and hence, may be important for macrophage regulation of T cell biology.

In summary, our findings provide new insight into human macrophage biology and establish the human placenta as an additional site of primitive haematopoiesis.

## Methods

Antibodies used, and their respective concentrations are listed in Supplementary Table 1, Other reagents, software and publicly available scRNAseq data used are listed in Supplementary Table 2.

### Patient samples and ethics

All tissue samples used were obtained with written consent from participants. First-trimester placental tissues were obtained from healthy women with apparently normal pregnancies undergoing elective first-trimester terminations (6–12 weeks estimated gestational age (EGA)). The EGA of the samples was determined from the last menstrual period. Estimations of post-conception weeks (PCW) ages for placental samples were obtained by subtracting 14 days from the EGA of each sample. Term placental samples were obtained from healthy women undergoing elective caesarean sections. Peripheral blood was taken from healthy adult volunteers. All samples were obtained with written informed consent from participants under ethical approval which was obtained from the Cambridge Research Ethics committee (study 04/Q0108/23).

### Tissue processing

Samples were processed immediately upon receipt as previously reported^2,55^. Samples were washed in PBS for 10 min with a stirrer before processing. The placental villi were scraped from the chorionic membrane with a scalpel and digested with 0.2% Trypsin (Pan-Biotech)/ 0.02% Ethylenediaminetetraacetic acid (EDTA) (Source BioScience) at 37 ^o^C with stirring, for 7 min (first-trimester samples) or 10 min (term samples). The digested cell suspension was passed through a sterile muslin gauze, and foetal bovine serum (FBS) (Sigma-Aldrich) was added to halt the digestion process. The undigested tissue left on the gauze was scraped off with a scalpel and digested in 2.5 ml 1 mg/ml collagenase V (Sigma-Aldrich), supplemented with 50 μl of 10 mg/ml DNAse I (Roche) for 20 min (first-trimester samples) or 45 min (term samples) at 37 ^o^C with agitation. The digested cell suspension was passed through a sterile muslin gauze and washed through with PBS. Cell suspensions from both the trypsin and collagenase digests were pelleted, resuspended in PBS and combined. Cells were layered onto a Pancoll gradient (PAN-biotech) and spun for 20 min without brake at 1741 g. The leukocyte layer was collected and washed in PBS. Blood samples were similarly layered onto a Pancoll gradient and processed as described previously^2,56^. Yolk sacs were washed in PBS, mechanically dissociated with scissors and digested in collagenase for 5 min, washed and filtered.

### Flow cytometry and data analysis

Cell suspensions were stained for viability with 1:1000 LIVE/DEAD Fixable Blue (Thermo Fisher Scientific), or 1:1000 Zombie Aqua (Biolegend), both for 20 min at 4 ^o^C, and washed twice in PBS. For cell sorting cell suspensions were stained for viability with 1:3000 4′,6-diamidino-2-phenylindole (DAPI) (Sigma-Aldrich) immediately before sorting.

Cells were blocked in human blocking buffer (5% human serum (Sigma-Aldrich), 1% rat serum (Sigma-Aldrich), 1% mouse serum (Sigma-Aldrich), 5% FBS and 2 mM EDTA) for 15 min at 4^o^C and were incubated with antibody cocktails for 30 min at 4^o^C. Antibodies used are listed in Supplementary Table 1. Cells were washed and resuspended in FACS buffer (PBS containing 2% FBS and 2 mM EDTA). The lineage (Lin) channel in flow cytometry analyses included combinations of the markers CD3, CD19, CD20, CD41, CD56, CD66b, CD235a and CD335, for the removal of contaminating T cells, B cells, NK cells erythrocytes, megakaryocytes/platelets and granulocytes. Flow cytometry was performed using a Cytek Aurora (Cytek) or an Attune NxT (Thermo Fisher Scientific), or cells were purified by cell-sorting using a BD FACS Aria III (BD bioscience). Flow cytometry data was analysed using FlowJo v10.7 (Treestar), R version3.6.3 (The R foundation) and Prism 9 (GraphPad).

The gating strategy for the isolation of PEMP, HBC and any intermediates was determined using the Hyperfinder plugin in FlowJo, using default parameters. Down-sampled gated cells were exported from FlowJo, imported into R and transformed using the ‘*autoLgcl’* transformation using the ‘*cytof_exprsMerge’* function from the Cytofkit2 R package. The datasets were batch-corrected using the ‘*mnnCorrect’* function from the batchelor R package and subjected to PCA analysis using the ‘*prcomp’* function.

Index-sort plate data was loaded into R using the ‘*retrieve_index’* function from the indexSort R package, and the resultant data matrices were transformed via the ‘*logicleTransform’* function in the flowCore R package (parameters; *w* = 0.6, *m* = 4.2, *a* = 0). Cells which generated colonies were manually selected and transformed marker expression data was extracted for visualisation and statistical analysis in Prism 9 (GraphPad).

### Cytospins of sort-purified cells

PEMP were sorted by FACS as detailed above. Cells from PEMP-derived colonies were taken for cytospins before they were stained for flow cytometry. Cells were resuspended in 150 μl of PBS and centrifuged onto glass slides using a Shandon Cytospin 2 (Marshall Scientific) at 10 g for 5 min. Cells were fixed in methanol for 2 min and air-dried. Slides were stained with 1:30 Giemsa-stain (Sigma-Aldrich) for 25 min and mounted in DePex mounting medium (BHD). Slides were imaged under a 63X objective on a Zeiss AxioObserver Z1 Microscope (Zeiss).

### Immunofluorescence of placental tissue sections

Sections from early first-trimester placenta villous tissue were prepared as described previously. Slides were placed in blocking buffer for 20 min at room temperature, washed in PBS and incubated overnight at 4 °C with primary antibodies. The slides were washed twice for 5 min in PBS, incubated with secondary antibodies for 1 h at room temperature and washed twice for 5 min in PBS. The slides were then stained with 1/1000 DAPI (Sigma-Aldrich) for 10 min at room temperature and washed twice for 5 min in PBS. Slides were mounted using ibidi mounting medium (ibidi). Slides were imaged using a Zeiss SP8 confocal LSM 700 (Zeiss). A description of the primary and secondary antibodies used is provided in Supplementary Table 1.

### Single cell differentiation assays

Primary placental fibroblasts were obtained by culturing placental digests in Dulbecco’s Modified Eagle Medium (DMEM) (Thermo Fisher Scientific) supplemented with 10% FBS, 2.5% Penicillin Streptomycin (Pen/Strep) (Sigma-Aldrich) and 20 μM L-Glutamine (Sigma-Aldrich). Non-adherent cells were removed, and cells were passaged a minimum of 4 times, yielding a pure population of fibroblasts, which were then cryopreserved. Placental fibroblasts were plated at a density of 3000 cells/well into flat 96-well plates in 100 μl DMEM (Thermo Fisher Scientific) supplemented with 10% FBS, 2.5% Pen/Strep (Sigma-Aldrich) and 20 μM L-Glutamine (Sigma-Aldrich) 2–3 days before sorting. On the day of the sort, the medium was changed to 100 μl/well StemPro-34 medium with nutrient supplement (Thermo Fisher Scientific) supplemented with cytokines (IL-6 10 ng/ml, IL-3 20 ng/ml, M-CSF 50 ng/ml and SCF 20 ng/ml; all Miltenyi Biotec), with 2.5% Pen/Strep and 20 μM L-Glutamine (Sigma-Aldrich). Cryopreserved placental and adult peripheral blood cells were used for single-cell differentiation assays, cells were thawed, washed, and stained for FACS as detailed above.

Cells were index sorted (1 cell/well) and cultured for a total of 18 days. On day 7, an additional 50 μl of media with cytokines was added, with the addition of EPO (Expex) and TPO (Miltenyi Biotec) to final concentrations of 1.5 units/ml and 25 ng/ml respectively. EPO and TPO were not added until day 7 to favour the differentiation of macrophages. Brightfield images of colonies were taken on an EVOS M5000 microscope (Thermo Fisher Scientific). After 18 days, plates containing colonies were incubated on ice for 15 min and colonies were removed from culture plates via gentle aspiration and washed in PBS. Colonies were then analysed by flow cytometry or cytospin, as detailed above. Lineage potentials were ascertained when the numbers of cells of the indicated phenotypes were more than 30 (for PEMP) or 20 (for GMP) per well.

### In vitro culture of Hofbauer cells

FACS isolated first-trimester HBC were cultured in flat-bottomed 96-well plates at a density of 100,000 cells/well, in 150μl ADMEM F12 (Gibco) supplemented with 10% FBS, 2.5% Pen/Strep (Sigma-Aldrich) and 20 μM L-Glutamine (Sigma-Aldrich) and 10 µg/ml M-CSF (Gibco). Media and cytokines were refreshed every three days. On Day 7, 14 and 30 of culture, cells were washed with PBS and removed from the plates by the addition of Accutase® (Biolegend) for 20 min. Cell viability was ~95% after removal from the plates. Cells were washed and stained as described above with DAPI and HLA-DR BV786.

### SmartSeq2 scRNAseq of placental cells

Live cells were isolated by FACS as detailed above and sorted into 96 well plates containing 2.3 μl of SmartSeq2 lysis buffer^57^ per well 10% SUPERase-ln RNase inhibitor (Thermo Fisher Scientific), 0.2% Triton X-100 (Sigma-Aldrich). Once cells were collected plates were sealed, spun at 700 g for 1 min and frozen using dry ice before storage at −80 °C. Sequencing libraries were constructed using the SmartSeq2 protocol^57^ and 150 BP paired-end sequencing was performed using a Illumina Novaseq SP.

### SmartSeq2 scRNAseq data pre-processing and visualisation

SmartSeq2 sequencing data were trimmed with Trim Galore (version 0.4.0, https://github.com/FelixKrueger/TrimGalore) default settings, low quality (Q < 20) and short reads (<20 bp) were removed. Remaining reads were then aligned with STAR (v 2.5.0.a)^58^ using the GRCh38 human reference genome and annotation, supplemented with External RNA Controls Consortium (ERCC) spike-in controls ‘ERCC92.fasta’ (https://gist.github.com/mikheyev/821f96a0ce76f58ee0ba). Gene-specific read counts were calculated using ‘featureCounts’ function in subread package (version 1.6.2)^59^.

Downstream analyses of gene expression matrices were performed using Seurat (v3.0)^60^. Cells with fewer than 3500 (Y028) or 2000 (Y054) detected genes, and more than 30% mitochondrial gene expression were removed, yielding a total of 255 cells. Samples were log-normalised and pre-processed individually following the Seurat v3 workflow. Clusters were identified in each dataset using the *FindNeighbours* and *FindClusters* functions in Seurat. Clusters were annotated on the basis of expression of known marker genes. For the purposes of this study 35 endothelial cells (*CDH5*^+^, *KDR*^+^) and 10 erythrocytes (*HBZ*^+^, *HBE1*^+^) were removed from the datasets. The two samples were merged and integrated via the Seurat v3 integration workflow. Clusters were identified using the *FindNeighbours* and *FindClusters* functions in Seurat. Uniform Manifold Approximation and Projection (UMAP) dimensionality reduction was performed using the *RunUMAP* function in Seurat, using the first 20 principal components (PCs). Significantly differentially expressed genes (DEGs) were identified using the *FindMarkers* function, using the Wilcox rank sum test, corrected for multiple comparisons. Pseudotime trajectory analysis of PEMP subsets was performed with the Slingshot R package^33^ and the calculated trajectory was overlain onto a diffusion map (DM) embedding, calculated using the *DiffusionMap* function in the Destiny R package^61^. Selected genes that varied across the Slingshot trajectories were plotted as heatmaps of smoothed normalised gene expression. Smoothing was performed using the *rollmean* function in the zoo R package

### Preparation of bisulphite-sequencing libraries

Low input bisulphite-sequencing libraries were generated using ≥5000 sorted cells, as previously described, using a post-bisulphite adaptor tagging (PBAT) method^62^. In brief, cells were thawed and then lysed in 10 mM Tris buffer with 0.5% SDS and proteinase K at 37 °C for 1 h. In a two-step process, first and second strand synthesis was performed using Klenow Fragment (3’->5’ exo-) enzyme (New England Biolabs) and customised 9 bp random sequence containing biotin-conjugated adaptors. Libraries were amplified using 10 amplification cycles with Phusion High-Fidelity DNA polymerase (New England Biolabs). Libraries were quantified using the High DNA Sensitivity Bioanalyzer 2500 (Agilent) and Illumina library quantification kit (KAPA). Samples were multiplexed and sequenced using 100 bp paired-end mode on the Illumina NovaSeq. A summary of the samples and sequencing depths is provided in Supplementary Table 3.

### Bisulphite sequencing data processing

PBAT libraries underwent quality and adaptor trimming using Trim Galore v0.4.5 with -clip parameters. Hits were filtered to remove mappings with a MAPQ score <20. Libraries were deduplicated and methylation calls extracted using PBAT mode in Bismark v0.16.3^63^ with paired-end alignment to the human GRCh38 genome assembly.

### Preparation of ATAC-sequencing libraries

Cells were FACS sorted into cold PBS and centrifuged for 5 min at 400 × g. Supernatants were carefully aspirated and cell pellets were resuspended in 100 μl ATAC-seq lysis buffer (made in house: 1 M Tris.Cl, 5 M NaCl, 1 M MgCl2, 20% Igepal in nuclease-free water) and incubated on ice for 10 min. Samples were centrifuged at 500 × g for 10 min and supernatants were aspirated carefully. Samples were resuspended in a 50 μl transposition reaction mix made up of the Illumina Tagment DNA Enzyme and Buffer Kit (Illumina), which consisted of TDE1 (tagment DNA enzyme) diluted in TD buffer and nuclease-free water, and incubated at 37 °C for 30 min. Following this, DNA isolation was carried out using a DNA concentration kit (Zymoresearch). DNA was eluted in 10 μl of elution buffer and stored at −20 °C until needed. Once all samples were collected, the NGS facility at the Wellcome – MRC Cambridge Stem Cell Institute amplified the library. Samples were subsequently sequenced on a NovaSeq 6000 System (Illumina) to yield an average of 30 million reads per sample.

### Analysis of publicly available scRNAseq data and generation of combined foetal scRNAseq datasets

Details and sources of publicly available datasets used in this study can be found in Supplementary Table 2 All datasets were processed as detailed above using Seurat. Datasets available as ‘.*h5ad’* format were converted into Seurat objects using the ‘*convert’* and ‘*LoadH5Seurat’* functions in the R package SeuratDisk. Where available, cluster annotations from the original studies were added to the meta-data of each object to allow for the identification of distinct cell types. Where annotations were not available clusters were manually annotated based on known marker genes, using the source publications as references.

For the construction of the early human embryo object (Fig. 5A) datasets from a CS7 Embryo (E-MTAB-9388, http://www.human-gastrula.net/)^25^, CS10 embro body (GSE135202)^26^, CS11 Caudal half (GSE135202)^26^ and CS11 yolk sac (GSE137010)^27^ were merged, and batch correction performed using the ‘*RunFastMNN’* function in the SeuratWrappers R package, correcting for the tissue of origin. Clustering and dimensionality reduction were performed using the first 20 MNN components, and clusters were annotated based on known marker gene expression.

The human foetal macrophage dataset (Fig. 5E, F, Supplementary Fig. 10A–D) comprised data from early human embryos (GSE133345)^27^, foetal microglia (GSE141862)^41^, foetal liver (E-MTAB-7407)^28^, foetal skin (E-MTAB-7407, and GSE179565)^28,39^, foetal gut (E-MTAB-8901, https://www.gutcellatlas.org/)^40^ and placenta (E-MTAB-6701)^24^. Each dataset was subset to include only macrophage populations. The early human embryo dataset was first subset by tissue of origin (head, liver or skin), and then subset to only include macrophage populations for each organ. As annotations were not available for the foetal skin datasets (GSE133345, E-MTAB-7407 and GSE179565), the original datasets were merged, batch-corrected and two major populations of *F13* *A1*^+^ and *CX3CR1*^+^ TRMs were identified via clustering and dimensionality reduction, consistent with source publications. All macrophage datasets were then merged, and batch correction performed using the ‘*RunFastMNN’* function correcting for the study each dataset was derived from. Dimensionality reduction was performed using the first 10 MNN components and macrophage populations were annotated based on both their identity and age for gene expression analysis. The proportion of macrophage populations expressing HLA Class II (Fig. 5E) was determined by calculating the proportion of cells co-expressing *HLA-DRA*, *HLA-DRB1*, *HLA-DPA1*, *HLA-DPB1*, *HLA-DQA1* and *HLA-DQB1*. Co-expression was defined as a cell exhibiting non-zero expression of all genes simultaneously.

The human foetal haematopoiesis dataset (Supplementary Fig. 3A) comprised data from a CS7 Embryo (E-MTAB-9388, http://www.human-gastrula.net/)^25^, CS10 embryo body (GSE135202)^26^, CS11 yolk sac (GSE137010)^27^, CS15 AGM (GSE135202)^26^, Foetal liver (E-MTAB-7407, https://www.covid19cellatlas.org/index.healthy.html)^28^, Foetal Femur (E-MTAB-9067, https://gitlab.com/cvejic-group/integrative-scrna-scatac-human-foetal/-/tree/master/Data/ScanpyObjets)^29^ and placenta (E-MTAB-6701)^24^. The foetal liver dataset was downsampled from 95,461 cells to 20,000 cells (maintaining the structure of the data) to ease computational burden. Datasets were merged, and batch correction performed using the ‘*RunFastMNN’* function correcting for the tissue of origin. From the resultant object haematopoietic cells were identified, subset and subjected to an additional round of batch correction, yielding the final object shown in the figure. A force-directed graph embedding was computed using the first 20 MNN components using ForceAtlas2 in Python 3 with 600 iterations.

### SCENIC analysis

To infer transcription factor activity in our scRNAseq data, gene regulatory network analysis was performed with SCENIC^30^. Regulons were inferred following the SCENIC analysis pipeline, and regulon activity scores for each cell were added as a new *Assay* to the scRNAseq Seurat object, permitting downstream analysis. Regulons with differential activity between clusters were calculated using *FindMarkers* function, and selected regulon activities were plotted using *pheatmap*. The regulonUMAP embedding was computed using regulon activity scores in the SCENIC-regulon assay of the Seurat object, with the first 15 principal components used.

### Comparisons of placental cells with an atlas of foetal liver haematopoiesis

Foetal liver haematopoiesis and placental scRNAseq datasets were processed as detailed above. To profile the transcriptomic similarity between these two datasets we followed the Seurat “Mapping and Annotating Query Datasets” (https://satijalab.org/seurat/articles/integration_mapping.html) vignette, with minor adjustments. The foetal liver dataset was used as the reference, and the placental scRNAseq data as the query. We first computed anchors between the query (placental) and reference (Foetal liver) datasets using the *FindTransferAnchors* function in Seurat, using the top 30 principal components. Foetal liver cluster prediction scores for each cell in the placental dataset were then calculated from these anchors using the TransferData function in Seurat. Higher prediction scores indicate a higher level of transcriptional similarity between a given query cell and a given reference cluster. Mean prediction scores for each placental scRNAseq cluster were plotted as a heatmap using *pheatmap*.

### Comparisons of PEMP-1 with other human foetal haematopoietic progenitors

Each individual progenitor population was subset from its original dataset; ‘CS7 EMP’ from CS7 Embryo (E-MTAB-9388, http://www.human-gastrula.net/)^25^, ‘YSMP’ from CS11-17 YS (GSE133345)^27^, ‘AGM HSC’ from CS15 AGM (GSE135202)^26^, ‘FL HSC’ and ‘BM HSC’ from foetal liver and foetal bone marrow (E-MTAB-9067, https://gitlab.com/cvejic-group/integrative-scrna-scatac-human-foetal/-/tree/master/Data/ScanpyObjets)^29^ and PEMP-1 from the scRNAseq dataset generated in this study. The datasets were merged, and integrated via the Seurat V3 integration workflow, correcting for the study of origin (Supplementary Fig. 7A, B). Transcriptomic similarity between progenitor populations was assessed using the ‘*BuildClusterTree’* function in Seurat utilising the 2000 features used for integration (Fig. 3B). For DEG analysis the object was down-sampled to 50 cells per population to prevent cell numbers confounding the analysis.

DEGs were identified between CS7 EMP and definitive progenitors, and PEMP-1 and definitive progenitors using the *FindMarkers* function with *logfc.threshold* = 0, using the Wilcox rank sum test, corrected for multiple comparisons. DEGs with a logFC >0.5 or < −0.5 and an adjusted *p*-value of <0.05 in either or both analyses were visualised.

### Comparisons of PEMP-1 with murine foetal haematopoietic progenitors

Murine placental data was obtained from GSE152903^37^ and murine FL, AGM and YS data was obtained from GSE137116^38^. To allow for the direct comparison of murine and human datasets we identified human homologs of murine genes from the gene expression count matrices, using the ‘*getLDS*’ function in the biomaRt R package. The murine count matrices were then subset to include only genes with direct human homologs, and murine gene annotations were replaced with the corresponding human gene annotation, to generate “humanised” murine scRNAseq data. “Humanised” murine data was pre-processed as detailed above and progenitor populations were identified either by manual data inspection (placental data) or provided annotations (FL, AGM ad YS data) and subset. Murine and human datasets were merged, and integrated via the Seurat v3 integration workflow, correcting for the species of origin (Supplementary Fig. 7C, D). Transcriptomic similarity between progenitor populations was assessed using the ‘*BuildClusterTree’* function in Seurat utilising the 2000 features used for integration (Fig. 3E). DEGs were identified between PEMP-1 and murine placental HSC using the *FindMarkers* function. DEGs were also calculated between all human and murine progenitor populations (all down-sampled to 50 cells) to allow for the identification of DEGs which are independent of the difference in species.

### DNA methylation analysis

CpG methylation files were loaded into SeqMonk software (v 1.48.0) for visualisation and analysis. Using autosomal 50 CpG running windows (*k* = 1,074,232), DNA methylation values were quantitated using the bisulphite-sequencing pipeline with a minimum coverage of 5 CpGs in each sample. 50-CpG windows overlapping the most upstream annotated transcription start site were assigned as promoters (*k* = 52,619). Differentially methylated regions (*k* = 710) were identified using logistic regression, using the default settings, and minimum 30% DNA methylation difference between first-trimester Hofbauer cells (*n* = 3) and third-trimester foetal monocytes (*n* = 3). Figures were generated in SeqMonk version 1.48.0, and boxplots were generated using ggplot2 in RStudio 2021.09.1.

### ATAC-seq analysis

Chromatin accessibility was quantified for 50 CpG windows overlapping the most upstream annotated transcription start site were assigned as promoters (*k* = 52,619), as defined above, using RPKM. Differentially methylated promoter 50 CpG windows (*k* = 710), as defined above, were compared to a set of random promoters (*k* = 200), of which 186 were informative for DNA methylation and ATAC-seq data. Fold change in chromatin accessibility was compared between first-trimester Hofbauer cells (*n* = 5) and third-trimester foetal monocytes (*n* = 5) across these domains using one-way ANOVA followed by Tukey’s post hoc tests, and at individual loci using a one-tailed unpaired *t*-test, correcting for multiple comparisons using Bonferroni correction. Boxplots were generated using ggplot2 in RStudio 2021.09.1.

### Statistical methods

Statistical tests of flow cytometry quantification data were performed using Prism 9 (Graphpad). *P* values for changes in abundance/expression were calculated by two-tailed Mann–Whitney tests. Correlation *p* values and R^2^ values were calculated by Pearson’s correlation tests. Differences in promoter DNA methylation between first trimester HBC and third trimester fetal monocytes were analysed by genome-wide logistic regression with Benjamini-Hochberg correction for multiple comparisons. Differences in chromatin accessibility between first trimester HBC and third trimester fetal monocytes were calculated via one-tailed *t*-test with correction for multiple comparisons using Bonferroni correction. Variance in promoter DNA methylation in placental myeloid cells across gestation and average difference in chromatin accessibility between first trimester HBCs and third trimester fetal monocytes at HBC hyper- and hypo-methylated and a random set of promoter 50 CpG windows were calculated by one-way ANOVA.

### Reporting summary

Further information on research design is available in the Nature Portfolio Reporting Summary linked to this article.

## Supplementary information

Supplementary Information

Reporting Summary

## Data Availability

The single-cell RNA, ATAC and bilsulphite sequencing data are available from the European Genome-phenome Archive (EGA) under accession number EGAS00001006981. These datasets are available under restricted access for compliance with ethics. The raw numbers for charts and graphs are included in the Source data file. Source data are provided with this paper.

## Source data

Source Data
