# Supplementary material for: Primitive haematopoiesis in the human placenta gives rise to macrophages with epigenetically silenced HLA-DR
Source: Nat Commun. 2023 Mar 30;14:1764. doi: 10.1038/s41467-023-37383-2 (PMC10063560; doi:10.1038/s41467-023-37383-2)
Supplement: Supplementary file 1 — Supplementary Information [file 41467_2023_37383_MOESM1_ESM.pdf]

Primitive haematopoiesis in the human placenta gives rise to macrophages with epigenetically silenced HLA-DR expression

Jake R. Thomas[1,2,5,] Anna Appios[1,2], Emily F. Calderbank[3], Nagisa Yoshida[1,2], Xiaohui Zhao[1], Russell S. Hamilton[1], Ashley Moffett[1,2], Andrew Sharkey[1,2], Elisa Laurenti[3], Courtney Hanna[1,4*], Naomi McGovern[1,2*]

[1]Centre for Trophoblast Research, University of Cambridge, UK
[2]Department of Pathology, University of Cambridge, UK
[3]Department of Haematology and Wellcome and MRC Cambridge Stem Cell Institute, University of Cambridge, Cambridge, UK
[4]Department of Physiology, Development and Neuroscience, University of Cambridge, UK
[5]Present Address: Life & Medical Sciences (LIMES) Institute, University of Bonn, Bonn, Germany

*Corresponding authors

Supplementary Materials

Figure S1

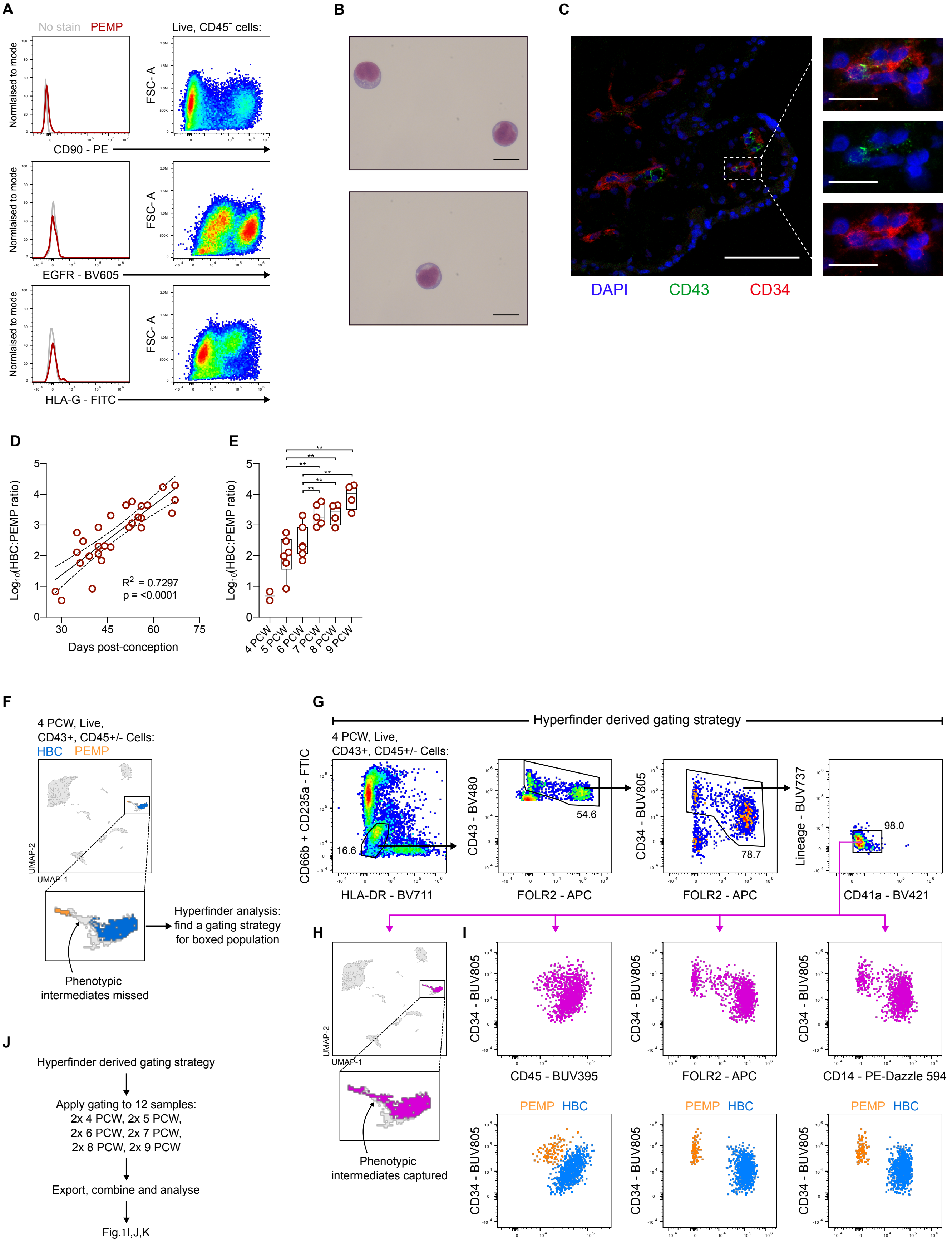
A
No stain PEMP
Live, CD45⁻ cells:
Normalised to mode
FSC-A
CD90 - PE
EGFR - BV605
HLA-G - FITC
B
C
DAPI CD43 CD34
D
Log10(HBC:PEMP ratio)
R2 = 0.7297
p = <0.0001
Days post-conception
E
4 PCW
5 PCW
6 PCW
7 PCW
8 PCW
9 PCW
F
4 PCW, Live,
CD43+, CD45+/- Cells:
HBC PEMP
UMAP-2
UMAP-1
Hyperfinder analysis:
find a gating strategy
for boxed population
Phenotypic
intermediates missed
G
Hyperfinder derived gating strategy
4 PCW, Live,
CD43+, CD45+/- Cells:
CD66b + CD235a - FTIC
HLA-DR - BV711
16.6
CD43 - BV480
FOLR2 - APC
54.6
CD34 - BUV805
78.7
Lineage - BUV737
CD41a - BV421
98.0
H
Phenotypic
intermediates captured
I
CD45 - BUV395
CD14 - PE-Dazzle 594
PEMP HBC
J
Hyperfinder derived gating strategy
Apply gating to 12 samples:
2x 4 PCW, 2x 5 PCW,
2x 6 PCW, 2x 7 PCW,
2x 8 PCW, 2x 9 PCW
Export, combine and analyse
Fig.1I,J,K

Fig.S1 Phenotypic analysis of PEMP. A) Analysis of CD90, EGFR and HLA-G expression in PEMP and Live, $CD45^-$ placental cells via flow cytometry, indicating that PEMP are not fibroblast or trophoblast contaminants. **B**) Giemsa-stained cytospins of PEMP, as used in Fig.1D. Scale bars, 20µm. Representative images of 3 independent experiments from *n* = 3 donors **C**) Identification of $CD34^+$ (red) $CD43^+$ (green) PEMP within the villous stroma of a 5 PCW placental sample. Scale bars, main panel 100µm, inset panels 20µm. Representative images of 4 independent experiments from *n* = 4 donors. **D**) Quantification of the ratio of HBC:PEMP determined by flow cytometry, plotted against post-conception age in days, or **E**) grouped by week. Boxplot centre lines represent the median, with box limits showing the upper and lower quartiles, and whiskers denoting minimum and maximum values. (*n* = 28 donors). (F-J) Development of a gating strategy for the isolation of HBC, PEMP and any phenotypic intermediates. **F**) UMAP visualisation of flow cytometry data from live $CD43^+$, $CD45^{+/-}$ cells from a 4 PCW, with gated HBC (Blue) and PEMP (Orange) overlain. Phenotypic intermediates between HBC and PEMP within UMAP embedding are missed by conventional gating. **G**) Hyperfinder-derived gating strategy for the isolation of HBC, PEMP and any phenotypic intermediates, and **H**) resultant gated population overlain onto original UMAP in pink. **I**) Phenotypic analysis of cells obtained from Hyperfinder-derived gating strategy (pink) compared to typical PEMP (orange) and HBC (blue) gating, revealing the capture of phenotypic intermediates. **J**) Analysis workflow for the use of the Hyperfinder-derived gating strategy for the generation of Fig.1K.

Figure S2

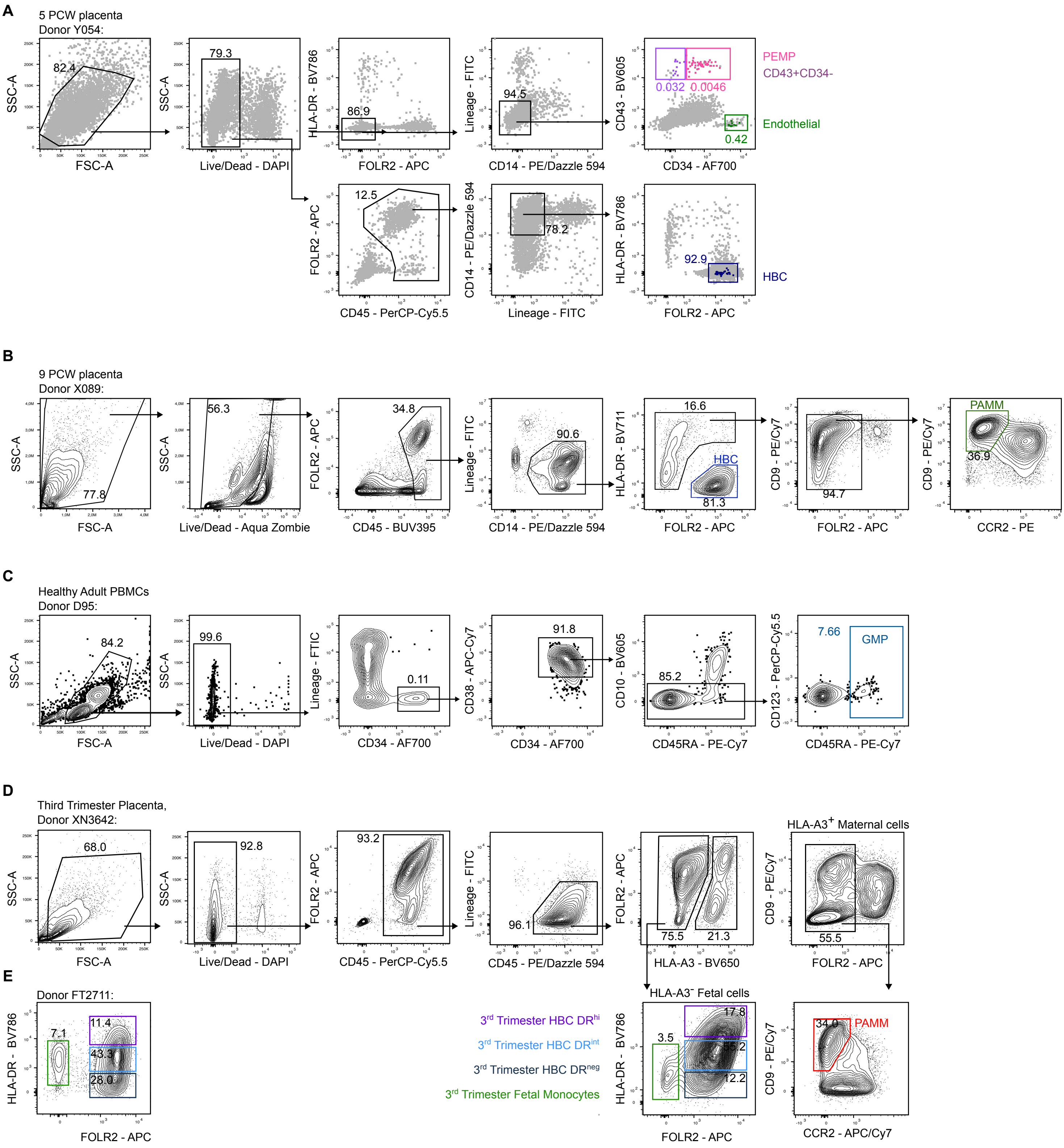
A
5 PCW placenta
Donor Y054:
SSC-A
FSC-A
82.4
79.3
Live/Dead - DAPI
86.9
HLA-DR - BV786
FOLR2 - APC
94.5
Lineage - FITC
CD14 - PE/Dazzle 594
CD43 - BV605
CD34 - AF700
0.032
0.0046
0.42
PEMP
CD43+CD34-
Endothelial
12.5
FOLR2 - APC
CD45 - PerCP-Cy5.5
78.2
CD14 - PE/Dazzle 594
Lineage - FITC
92.9
HLA-DR - BV786
FOLR2 - APC
HBC
B
9 PCW placenta
Donor X089:
77.8
56.3
Live/Dead - Aqua Zombie
34.8
CD45 - BUV395
90.6
16.6
HLA-DR - BV711
HBC
81.3
CD9 - PE/Cy7
94.7
PAMM
36.9
CCR2 - PE
C
Healthy Adult PBMCs
Donor D95:
84.2
99.6
Lineage - FTIC
0.11
CD34 - AF700
CD38 - APC-Cy7
91.8
CD10 - BV605
85.2
CD45RA - PE-Cy7
CD123 - PerCP-Cy5.5
7.66
GMP
D
Third Trimester Placenta,
Donor XN3642:
68.0
92.8
93.2
CD45 - PerCP-Cy5.5
96.1
CD45 - PE/Dazzle 594
75.5
21.3
HLA-A3 - BV650
HLA-A3+ Maternal cells
55.5
E
Donor FT2711:
7.1
11.4
43.3
28.0
3rd Trimester HBC DRhi
3rd Trimester HBC DRint
3rd Trimester HBC DRneg
3rd Trimester Fetal Monocytes
HLA-A3- Fetal cells
3.5
17.8
55.2
12.2
34.0
PAMM
CCR2 - APC/Cy7

Fig.S2 Gating strategies used for the isolation of cell populations. A) Full representative flow cytometry gating strategy for the isolation of PEMP, $CD43^{+}CD34^{-}$ cells, endothelial cells and HBC from first trimester placental digests for phenotyping, functional analyses and plate-based SmartSeq2 scRNAseq. Related to Fig.1B,C,D, Fig2A, Fig.4, Fig.S1A,B, FigS.5D, Fig.S6, Fig.S8G,H and Fig.S11 **B**) Full representative flow cytometry gating strategy for the isolation of HBC and PAMM (placenta-associated maternal macrophages) for phenotyping and functional analyses. Related to Fig.1C, Fig.6, Fig.S1D,E. **C**) Full representative flow cytometry gating strategy for the isolation of healthy adult blood GMP (Granulo-myeloid progenitors) for single cell differentiation assays. Data representative of n = 3 donors. Related to Fig.4 and Fig.S8I,J,K. **D**) Full representative flow cytometry gating strategy for the isolation of HBC subsets, foetal monocytes and PAMM from third trimester placental digests for bisulphite sequencing and ATACseq. Related to Fig.6 and Fig.S11. **E**) Representative FACS plot of foetal myeloid cells from a distinct donor, displaying the gating strategy for the isolation of HBC subsets and foetal monocytes, as in Fig.6.A.

Figure S3

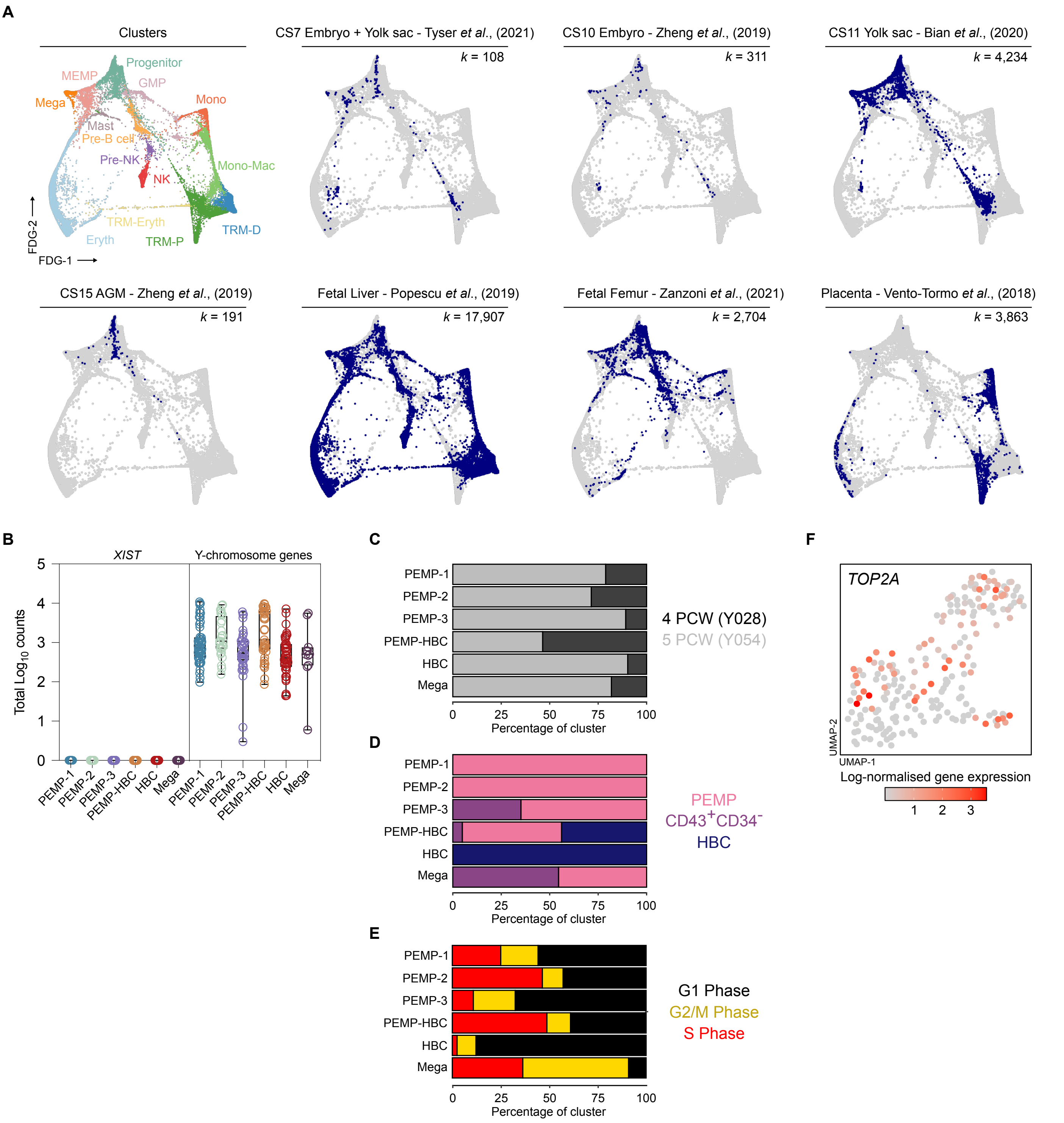

A
Clusters
Progenitor
MEMP
GMP
Mega
Mono
Mast
Pre-B cell
Pre-NK
Mono-Mac
NK
TRM-Eryth
TRM-D
Eryth
TRM-P
FDG-2
FDG-1
CS7 Embryo + Yolk sac - Tyser et al., (2021)
k = 108
CS10 Embyro - Zheng et al., (2019)
k = 311
CS11 Yolk sac - Bian et al., (2020)
k = 4,234
CS15 AGM - Zheng et al., (2019)
k = 191
Fetal Liver - Popescu et al., (2019)
k = 17,907
Fetal Femur - Zanzoni et al., (2021)
k = 2,704
Placenta - Vento-Tormo et al., (2018)
k = 3,863
B
XIST
Y-chromosome genes
Total Log10 counts
PEMP-1
PEMP-2
PEMP-3
PEMP-HBC
HBC
Mega
C
4 PCW (Y028)
5 PCW (Y054)
Percentage of cluster
D
PEMP
CD43+CD34-
HBC
E
G1 Phase
G2/M Phase
S Phase
F
TOP2A
UMAP-2
UMAP-1
Log-normalised gene expression

Fig.S3 scRNAseq of PEMP from early placental samples. A) Force-directed graph embedding of 29,318 human haematopoietic cells from a range of anatomical sites and developmental timepoints. Data derived from[21–26]. Top left panel – cells are coloured by their cluster identity. Other panels - cells from each study are highlighted in blue within each panel. Number of cells from each dataset (*k*) are annotated on each panel. GMP – Granulo-myeloid progenitor, MEMP – Megakaryocyte-Erythroid-Mast cell progenitor, Mega – Megakaryocyte progenitor, Eryth -Erythroid cells, Mono – Monocytes, Mono-Mac – Monocyte-derived macrophage, TRM-D – Definitive tissue-resident macrophages, TRM-P – Primitive tissue-resident macrophages, NK – Natural Killer cells. **B**) Boxplots showing the log-normalised total counts for *XIST* and for all genes from the Y-chromosome for each cluster in the scRNAseq dataset. XIST expression was not detected in any cell in the dataset, and all cells displayed Y-chromosome-specific counts, suggesting no contamination of maternal cells, and that all profiled cells are foetal in origin. Boxplot centre lines represent the median, with box limits showing the upper and lower quartiles, and whiskers denoting minimum and maximum values. (C-E) Stacked bar charts indicating the proportion of cells from each cluster **C**) derived from each donor, **D**) derived from each gate during FACS isolation or **E**) predicted to be in each stage of the cell cycle, as determined by cell cycle scoring. **F**) UMAP visualisation of scRNAseq data with heatmap overlay of TOP2A log-normalised gene expression.

Figure S4

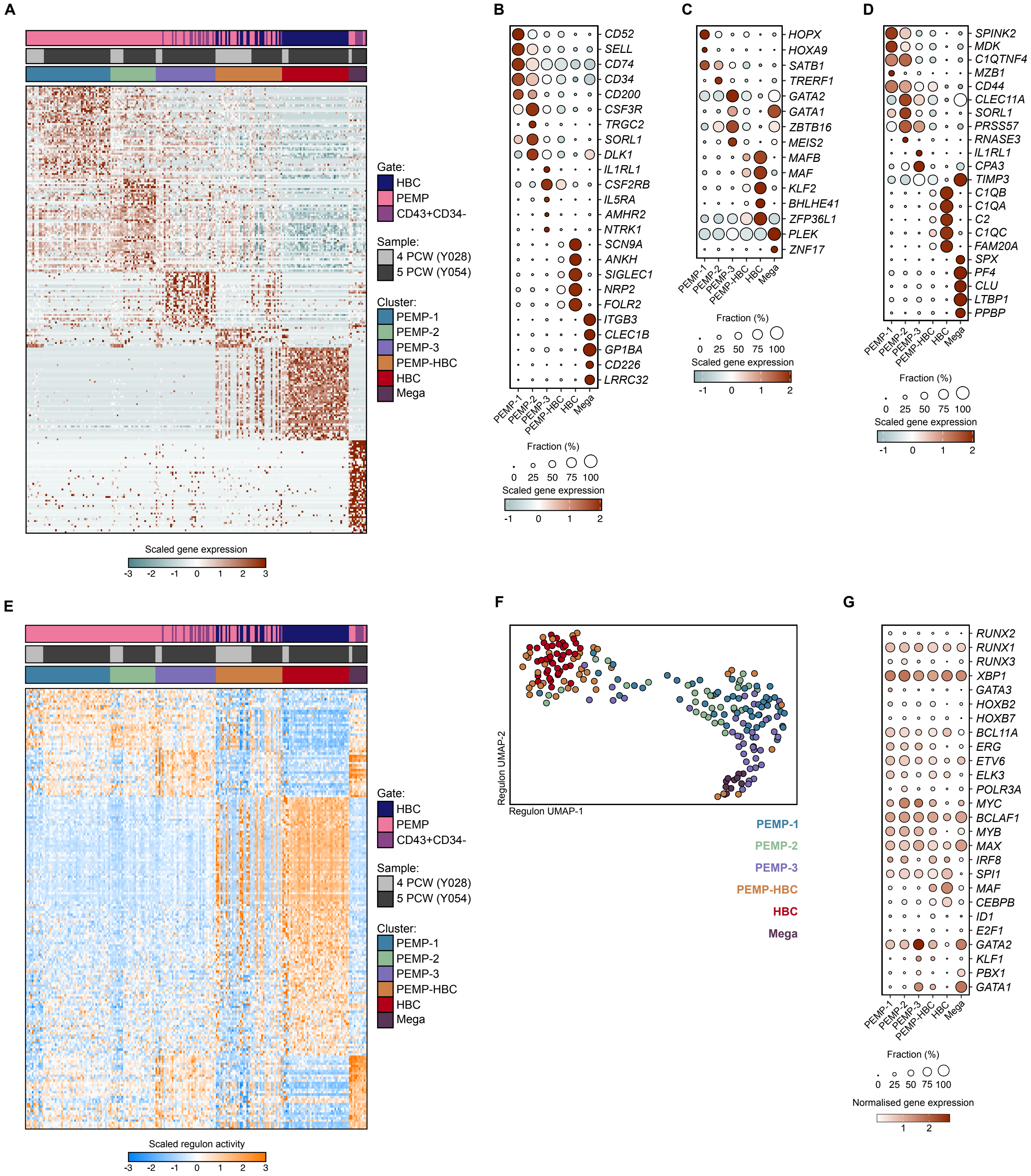
A
B
C
D
E
F
G
Gate:
HBC
PEMP
CD43+CD34-
Sample:
4 PCW (Y028)
5 PCW (Y054)
Cluster:
PEMP-1
PEMP-2
PEMP-3
PEMP-HBC
HBC
Mega
Scaled gene expression
-3 -2 -1 0 1 2 3
CD52
SELL
CD74
CD34
CD200
CSF3R
TRGC2
SORL1
DLK1
IL1RL1
CSF2RB
IL5RA
AMHR2
NTRK1
SCN9A
ANKH
SIGLEC1
NRP2
FOLR2
ITGB3
CLEC1B
GP1BA
CD226
LRRC32
HOPX
HOXA9
SATB1
TRERF1
GATA2
GATA1
ZBTB16
MEIS2
MAFB
MAF
KLF2
BHLHE41
ZFP36L1
PLEK
ZNF17
SPINK2
MDK
C1QTNF4
MZB1
CD44
CLEC11A
SORL1
PRSS57
RNASE3
IL1RL1
CPA3
TIMP3
C1QB
C1QA
C2
C1QC
FAM20A
SPX
PF4
CLU
LTBP1
PPBP
Fraction (%)
0 25 50 75 100
Scaled gene expression
-1 0 1 2
Scaled regulon activity
-3 -2 -1 0 1 2 3
Regulon UMAP-2
Regulon UMAP-1
RUNX2
RUNX1
RUNX3
XBP1
GATA3
HOXB2
HOXB7
BCL11A
ERG
ETV6
ELK3
POLR3A
MYC
BCLAF1
MYB
MAX
IRF8
SPI1
MAF
CEBPB
ID1
E2F1
GATA2
KLF1
PBX1
GATA1
Normalised gene expression
1 2

Fig.S4 Differential gene expression and transcription factor activity across scRNAseq clusters. **A**) Heatmap showing scaled log-normalised gene expression of the top 50 differentially expressed genes per cluster from the scRNAseq dataset. (B-D) Dotplot heatmap displaying scaled log-normalised gene expression of the top 5 **B**) surface markers **C**) transcription factors and **D**) secreted factors per scRNAseq cluster. Dot size represents fraction of cells with nonzero expression. **E**) Heatmap showing scaled inferred transcription factor activity (regulon scores) of all regulons with differential activity across clusters from the scRNAseq dataset. **F**) UMAP visualisation of placental scRNAseq dataset with embeddings calculated using regulon scores instead of gene expression data. **G**) Dotplot heatmap displaying log-normalised gene expression of transcription factors identified by SCENIC analysis across scRNAseq clusters.

Figure S5

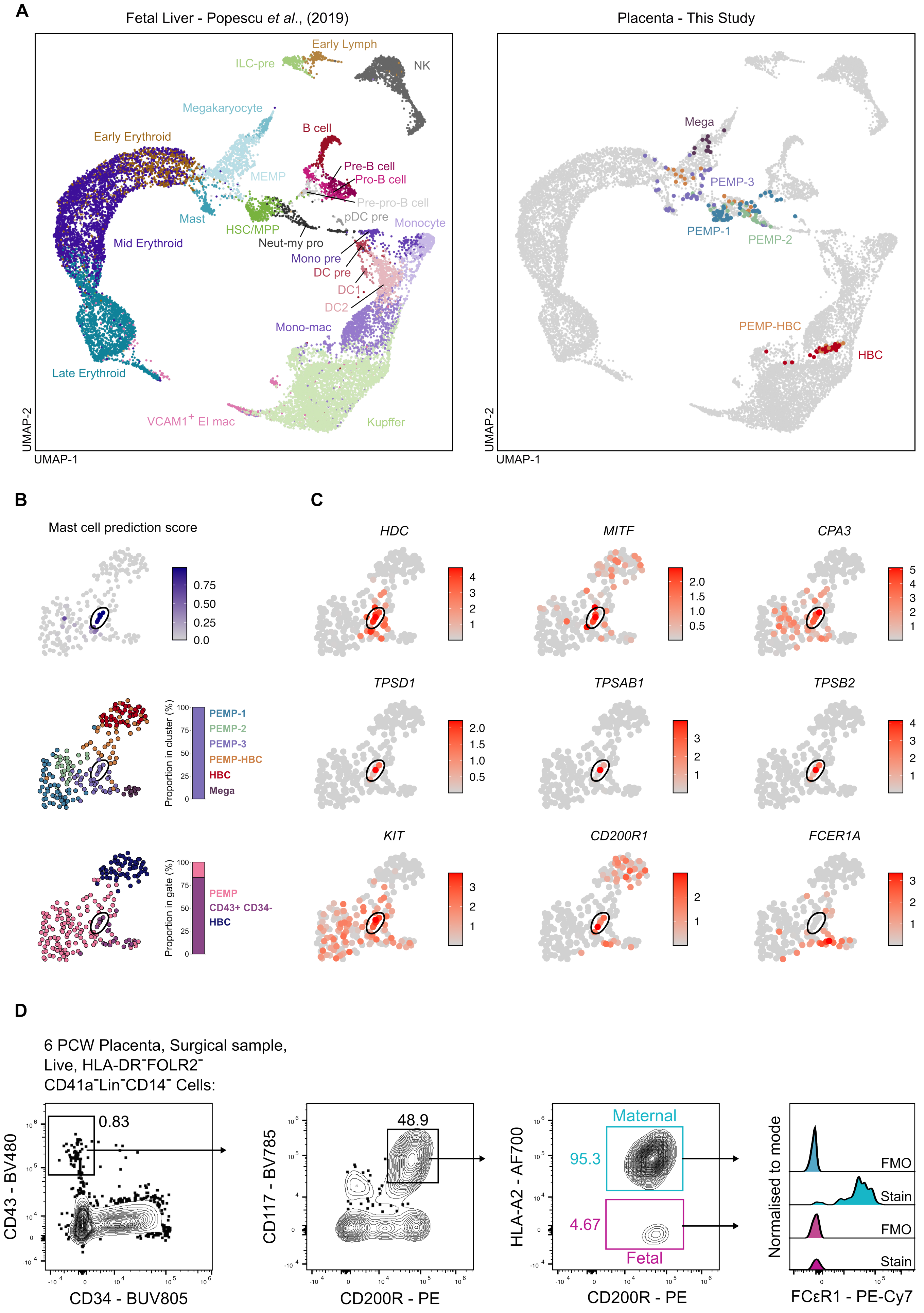

A
Fetal Liver - Popescu et al., (2019)
Placenta - This Study
Early Lymph
ILC-pre
NK
Megakaryocyte
B cell
Early Erythroid
Pre-B cell
Pro-B cell
MEMP
Pre-pro-B cell
Mast
pDC pre
Monocyte
HSC/MPP
Mid Erythroid
Neut-my pro
Mono pre
DC pre
DC1
DC2
Mono-mac
Late Erythroid
VCAM1+ EI mac
Kupffer
UMAP-2
UMAP-1
Mega
PEMP-3
PEMP-1
PEMP-2
PEMP-HBC
HBC
B
Mast cell prediction score
0.75
0.50
0.25
0.0
Proportion in cluster (%)
PEMP-1
PEMP-2
PEMP-3
PEMP-HBC
HBC
Mega
Proportion in gate (%)
PEMP
CD43+ CD34-
HBC
C
HDC
MITF
CPA3
TPSD1
TPSAB1
TPSB2
KIT
CD200R1
FCER1A
D
6 PCW Placenta, Surgical sample,
Live, HLA-DR⁻FOLR2⁻
CD41a⁻Lin⁻CD14⁻ Cells:
0.83
CD43 - BV480
CD34 - BUV805
48.9
CD117 - BV785
CD200R - PE
Maternal
95.3
4.67
Fetal
HLA-A2 - AF700
CD200R - PE
Normalised to mode
FMO
Stain
FMO
Stain
FCεR1 - PE-Cy7

Fig.S5 Dataset integration and placental foetal **mast cells. A**) Left, UMAP visualisation of foetal liver scRNAseq data[25], after *mnnCorrect* integration with placental scRNAseq dataset. Right, UMAP visualisation of placenta scRNAseq data, after *mnnCorrect* integration with foetal liver scRNAseq dataset. **B**) UMAP visualisation of placental scRNAseq dataset with foetal liver mast cell prediction score from Fig.2D overlain. Cells with high mast cell prediction scores are circled, and the proportions of these cells in each cluster and FACS gate are indicated in the stacked bar charts in the lower panels. **C**) UMAP visualisation of placental scRNAseq dataset with overlays of log-normalised expression of mast cell-specific genes. Cells with high mast cell prediction scores are circled. foetal placental mast cells lack the expression of *FCER1A* consistent with previous findings[28,29]. **D**) Identification of maternal $CD200R^+CD117^+FC\varepsilon R1^+$ and foetal $CD200R^+CD117^+ FC\varepsilon R1^-$ mast cells from a first trimester placenta surgical sample via flow cytometry. Maternal mast cells are likely derived from contamination of the maternal decidua, which is more prevalent in surgical samples. FMO – Fluorescence minus one.

Figure S6

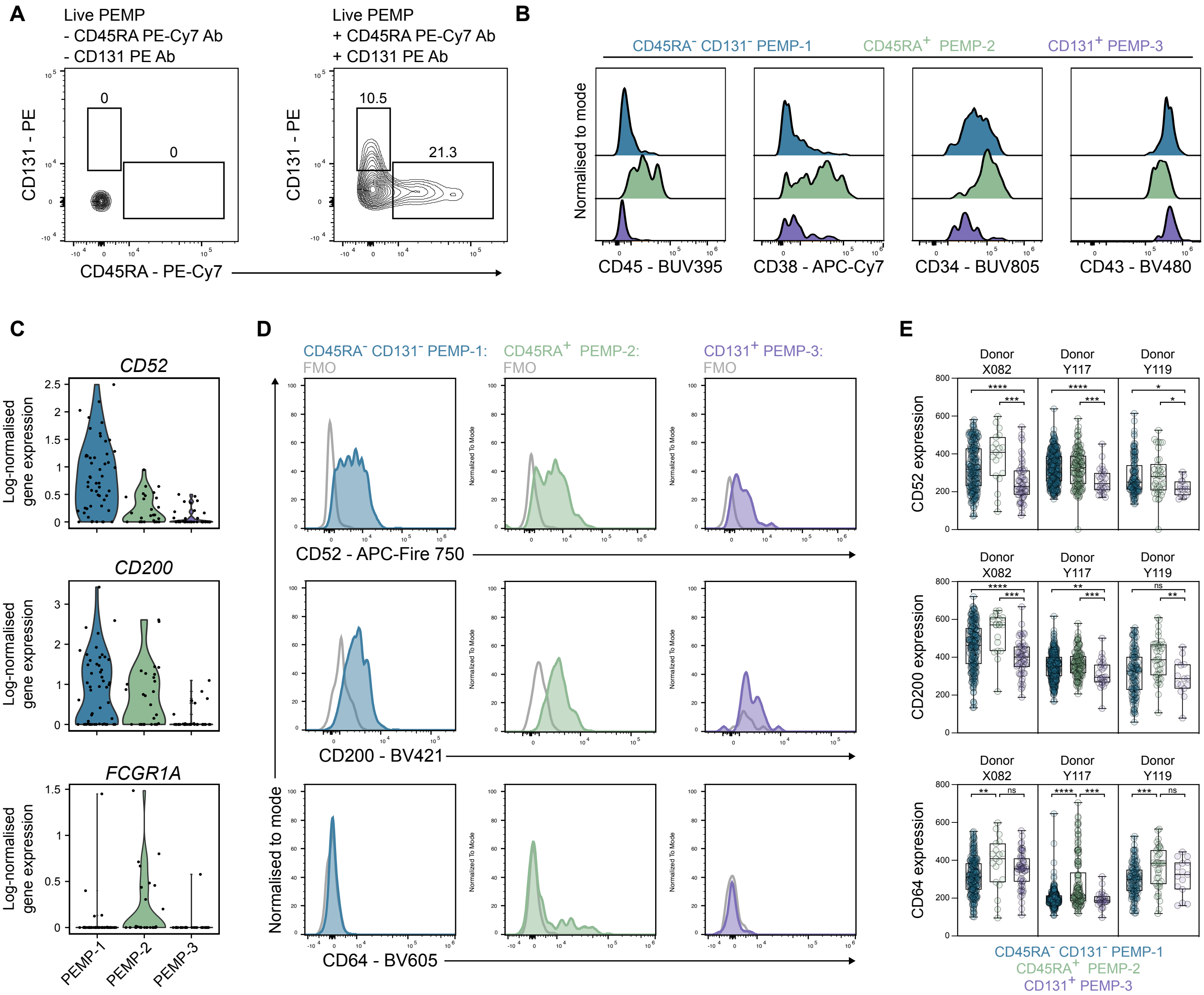
A
Live PEMP
- CD45RA PE-Cy7 Ab
- CD131 PE Ab
Live PEMP
+ CD45RA PE-Cy7 Ab
+ CD131 PE Ab
CD131 - PE
CD45RA - PE-Cy7
0
0
10.5
21.3
B
CD45RA⁻ CD131⁻ PEMP-1
CD45RA⁺ PEMP-2
CD131⁺ PEMP-3
Normalised to mode
CD45 - BUV395
CD38 - APC-Cy7
CD34 - BUV805
CD43 - BV480
C
CD52
CD200
FCGR1A
Log-normalised gene expression
PEMP-1
PEMP-2
PEMP-3
D
CD45RA⁻ CD131⁻ PEMP-1:
FMO
CD45RA⁺ PEMP-2:
FMO
CD131⁺ PEMP-3:
FMO
Normalised to mode
CD52 - APC-Fire 750
CD200 - BV421
CD64 - BV605
E
Donor X082
Donor Y117
Donor Y119
CD52 expression
CD200 expression
CD64 expression
CD45RA⁻ CD131⁻ PEMP-1
CD45RA⁺ PEMP-2
CD131⁺ PEMP-3

Fig.S6 Phenotypic analysis of PEMP. A) Analysis of CD131 and CD45RA expression within PEMP via flow cytometry without CD131 and CD45RA antibodies added (left panel) and with antibodies added (right panel). **B**) Analysis of CD45, CD38, CD34 and CD43 expression in $CD45RA^{-}CD131^{-}$ PEMP-1 (blue), $CD45RA^{+}$ PEMP-2 (green) and $CD131^{+}$ PEMP-3 (purple) via flow cytometry. Data shown are representative of *n* = 4 donors. **C**) Violin plots of *CD52*, *CD200* and *FCGR1A* gene expression across PEMP subsets from scRNAseq data. **D**) Representative flow cytometry histograms showing CD52, CD200 and CD64 expression in $CD45RA^{-}CD131^{-}$ PEMP-1 (blue), $CD45RA^{+}$ PEMP-2 (green) and $CD131^{+}$ PEMP-3 (purple) relative to FMO negative control. Data shown are representative of 3 experiments (*n* = 3 donors). **E**) Quantification of CD52, CD200 and CD64 expression for all cells in each PEMP gate for a total of 3 donors. Boxplot centre lines represent the median, with box limits showing the upper and lower quartiles, and whiskers denoting minimum and maximum values**.** All P values calculated by Mann-Whitney test. *, $P \leq 0.05$, **, $P \leq 0.01$; ***, $P \leq 0.001$, ****, $P \leq 0.0001$. ns, $P > 0.05$.

Figure S7

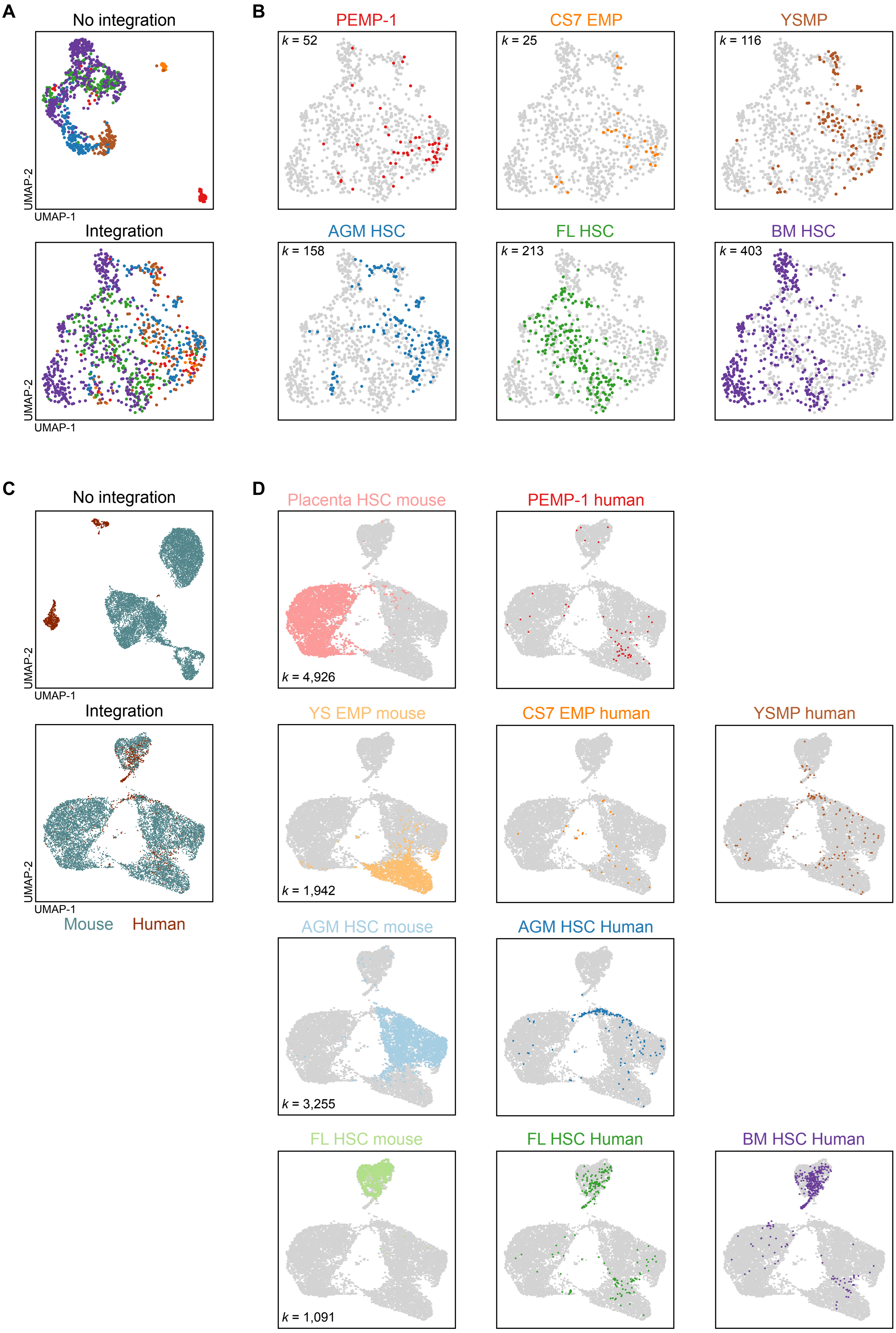
A
No integration
UMAP-2
UMAP-1
Integration
UMAP-2
UMAP-1
B
PEMP-1
k = 52
CS7 EMP
k = 25
YSMP
k = 116
AGM HSC
k = 158
FL HSC
k = 213
BM HSC
k = 403
C
No integration
UMAP-2
UMAP-1
Integration
UMAP-2
UMAP-1
Mouse
Human
D
Placenta HSC mouse
k = 4,926
PEMP-1 human
YS EMP mouse
k = 1,942
CS7 EMP human
YSMP human
AGM HSC mouse
k = 3,255
AGM HSC Human
FL HSC mouse
k = 1,091
FL HSC Human
BM HSC Human

Fig.S7 Intra- and inter-species foetal **haematopoietic progenitor datasets. A**) UMAP visualisations of 967 single cells from human haematopoietic progenitor populations used for comparative analysis (Fig.3B)[22–24,26], shown before (upper panel) and after (lower panel) Seurat V3 integration[53], correcting for the study of origin of each cell. Cells are coloured according to identity, see panel B. **B**) UMAP visualisation of cells of each identity in individual panels. Number of cells of each identity (*k*) are annotated on each panel. CS – Carnegie Stage, EMP – Erythro-myeloid progenitors, YSMP – Yolk sac myeloid-biased progenitors, AGM – Aorta-gonad mesonephros, FL – Foetal liver, BM – Bone marrow, HSC – Haematopoietic stem cell. **C**) UMAP visualisations of 12,181 human and murine foetal haematopoietic progenitors used for comparative analysis[22–24,26,34,35] shown before (upper panel) and after (lower panel) Seurat V3 integration[53], correcting for the species of each cell. Cells are coloured according to their species. Mouse data were "humanised" for this analysis by replacing murine genes with direct human homologs (see methods). **D**) UMAP visualisation of cells of each identity in individual panels. Number of murine cells of each identity (*k*) are annotated on each panel. YS – Yolk sac.

Figure S8

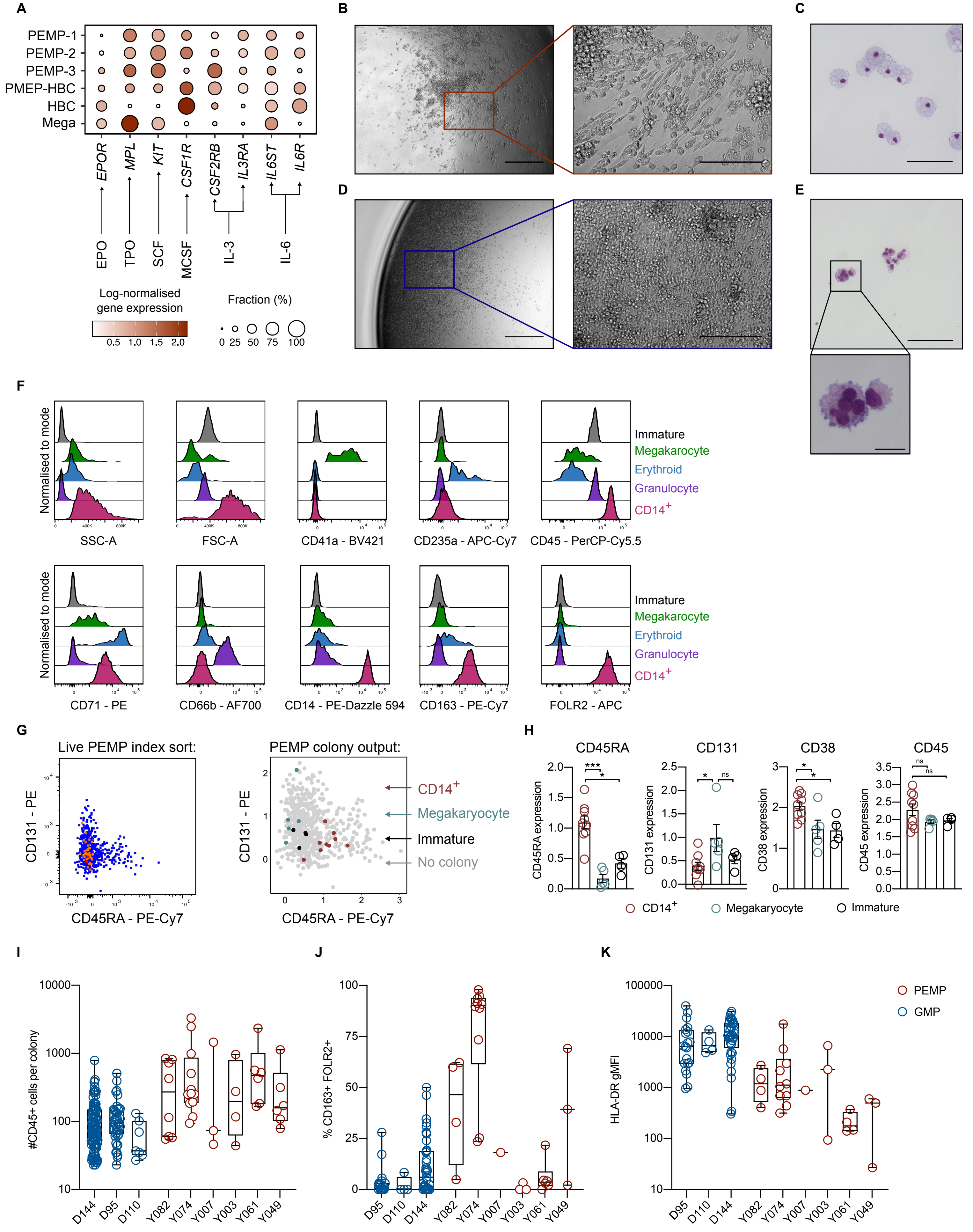

A
B
C
D
E
F
G
H
I
J
K
PEMP-1
PEMP-2
PEMP-3
PMEP-HBC
HBC
Mega
EPOR
MPL
KIT
CSF1R
CSF2RB
IL3RA
IL6ST
IL6R
EPO
TPO
SCF
MCSF
IL-3
IL-6
Log-normalised gene expression
0.5 1.0 1.5 2.0
Fraction (%)
0 25 50 75 100
Normalised to mode
Immature
Megakaryocyte
Erythroid
Granulocyte
CD14+
SSC-A
FSC-A
CD41a - BV421
CD235a - APC-Cy7
CD45 - PerCP-Cy5.5
CD71 - PE
CD66b - AF700
CD14 - PE-Dazzle 594
CD163 - PE-Cy7
FOLR2 - APC
Live PEMP index sort:
PEMP colony output:
CD131 - PE
CD45RA - PE-Cy7
No colony
CD45RA
CD131
CD38
CD45
CD45RA expression
CD131 expression
CD38 expression
CD45 expression
#CD45+ cells per colony
% CD163+ FOLR2+
HLA-DR gMFI
PEMP
GMP
D144
D95
D110
Y082
Y074
Y007
Y003
Y061
Y049

Fig.S8 PEMP single-cell differentiation assays. **A**) Dotplot heatmap displaying log-normalised gene expression of cytokine receptor genes within the placental scRNAseq dataset. Cognate ligands which are components of the PEMP differentiation cytokine cocktail are annotated onto the plot with arrows. Dot size represents fraction of cells with nonzero expression. **B**) Representative morphology of a $CD14^+$ colony derived from a single PEMP after 18 days of culture on primary placental fibroblasts. Scale bars, 500µm left panel, 100µm right panel. Representative image from *n* = 6 donors. **C**) Representative Giemsa-stained cytospins of cells from a $CD14^+$ colony derived from a single PEMP. Scale bars, 100µm. Representative image from *n* = 4 donors. **D**) Representative morphology of a diverse haematopoietic colony derived from a single PEMP after 18 days of culture on primary placental fibroblasts. Scale bars, 500µm left panel, 100µm right panel. Representative image from *n* = 6 donors. **E**) Representative Giemsa-stained cytospins of cells from a diverse haematopoietic colony derived from a single PEMP. Inset, morphology of a suspected PEMP-derived polyploid megakaryocyte. The colony from which this cytospin was taken was found to have made megakaryocytes via flow cytometry analysis. Scale bars, 100µm left panel, 20µm right panel. Representative image from *n* = 4 donors. **F**) Comparative analysis of marker expression via flow cytometry in distinct immune lineages derived from PEMP. **G**) Flow cytometry analysis of CD45RA and CD131 expression in 576 index-sorted PEMP from *n* = 2 donors (left panel), and colony output from resulting cultures (see Fig.4.D,E for colony gating strategy) overlain onto logicle-transformed biaxial plot of CD45RA and CD131 expression. Annotated colonies are gated as in Fig.4D,E: $CD14^+$ – Live, $CD45^+CD14^+CD66b^-$, Megakaryocyte – Live, $CD41a^+$, Immature – Live, $CD45^+$. **H**) Quantification of the expression of CD45RA, CD131, CD38 and CD45 by index-sorted PEMP which give rise to macrophage, megakaryocyte and immature colonies. P-values calculated by two-tailed Mann-Whitney test. Flow cytometry quantification data is represented by mean ± SEM. Exact p values: CD45RA:

CD14+ vs Megakaryocyte = 0.0010, CD14+ vs Immature = 0.0112. CD131: CD14+ vs Megakaryocyte = 0.0120, Megakaryocyte vs Immature = 0.1111. CD38: CD14+ vs Megakaryocyte = 0.0290, CD14+ vs Immature = 0.0196. CD45: CD14+ vs Megakaryocyte = 0.4376, CD14+ vs Immature = 0.6042. (H-J) Boxplot quantifications of **I**) number of $CD45^+$ cells per colony(GMP *n* = 3 donors, *k* = 157 colonies) (PEMP *n* = 6 donors, *k* = 39 colonies), **J**) percentage co-expression of CD163 and FOLR2 expression in $CD14^+$ colonies (GMP *n* = 3 donors, *k* = 61 colonies) (PEMP *n* = 6 donors, *k* = 25 colonies) and **K**) HLA-DR expression in CD14+ colonies (GMP *n* = 3 donors, *k* = 61 colonies) (PEMP *n* = 6 donors, *k* = 25 colonies), for each donor profiled. Boxplot centre lines represent the median, with box limits showing the upper and lower quartiles, and whiskers denoting minimum and maximum values. ns, $P > 0.05$, *, $P \leq 0.05$, ***, $P \leq 0.001$.

Figure S9

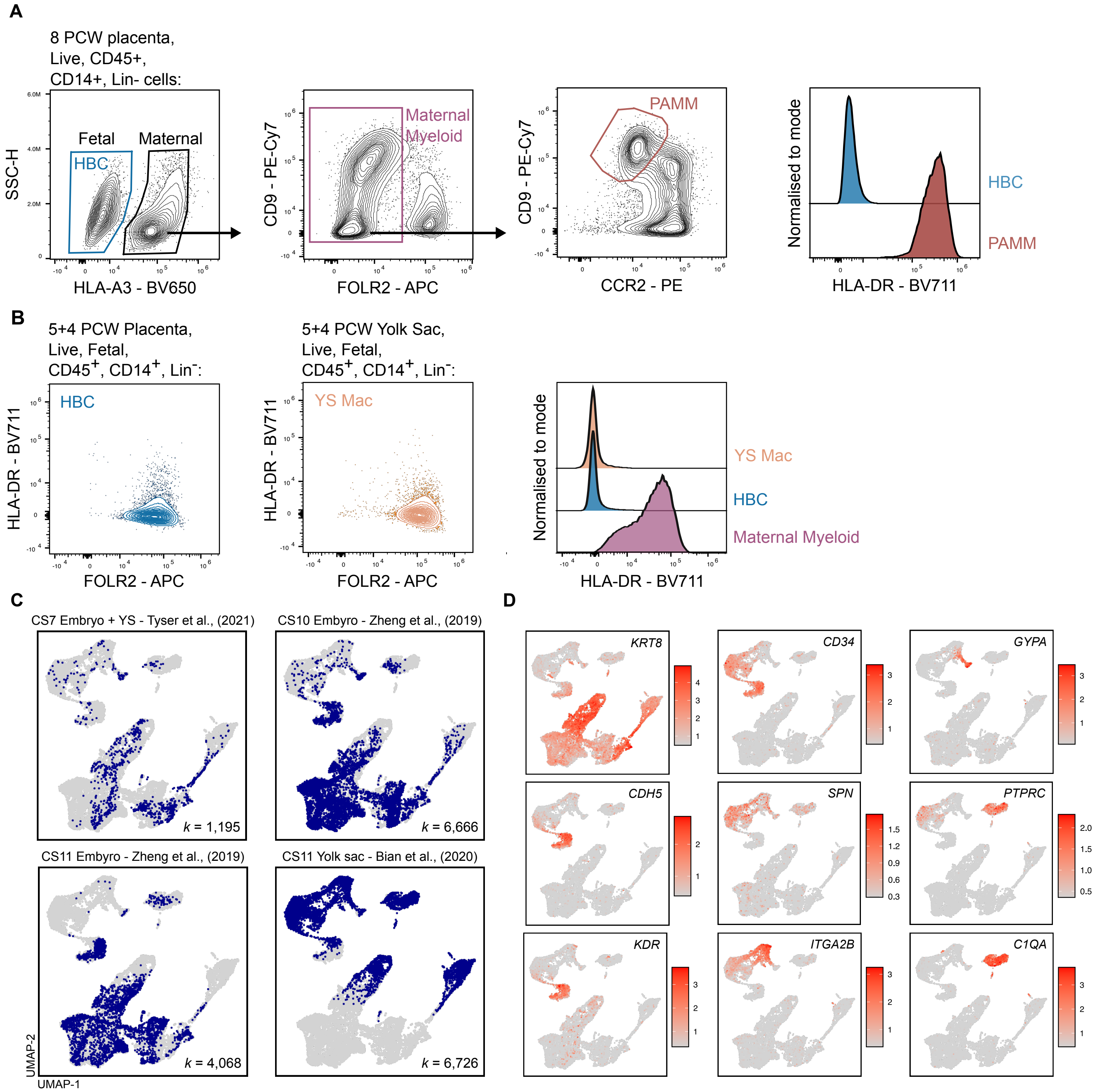
A
8 PCW placenta, Live, CD45+, CD14+, Lin- cells:
Fetal
Maternal
HBC
SSC-H
HLA-A3 - BV650
CD9 - PE-Cy7
FOLR2 - APC
Maternal Myeloid
PAMM
CCR2 - PE
Normalised to mode
HLA-DR - BV711
HBC
PAMM
B
5+4 PCW Placenta, Live, Fetal, CD45+, CD14+, Lin-:
5+4 PCW Yolk Sac, Live, Fetal, CD45+, CD14+, Lin-:
HBC
YS Mac
HLA-DR - BV711
FOLR2 - APC
Normalised to mode
YS Mac
HBC
Maternal Myeloid
HLA-DR - BV711
C
CS7 Embryo + YS - Tyser et al., (2021)
CS10 Embyro - Zheng et al., (2019)
k = 1,195
k = 6,666
CS11 Embyro - Zheng et al., (2019)
CS11 Yolk sac - Bian et al., (2020)
k = 4,068
k = 6,726
UMAP-2
UMAP-1
D
KRT8
CD34
GYPA
CDH5
SPN
PTPRC
KDR
ITGA2B
C1QA

Fig.S9 HBC HLA-DR expression and early human embryonic scRNAseq analysis. A) Representative flow cytometry gating strategy identifying foetal HBC (blue), and maternal PAMM1a – Placenta-associated maternal macrophages (red), and flow cytometry analysis of HLA-DR expression in HBC and PAMM. **B**) Flow cytometric analysis of FOLR2 and HLA-DR expression in donor-matched placenta HBC (blue), placental maternal myeloid cells (macrophages and monocytes) (purple) and yolk sac macrophages (orange). Histogram illustrates surface HLA-DR expression (A,B). **C**) UMAP visualisation of human early embryo single-cell transcriptomes, with cells from each study highlighted in blue within each panel. Number of cells from each dataset (*k*) are annotated on each panel. **D**) UMAP visualisation with heatmap overlays of log-normalised expression of key marker genes for the identification of different lineages. *KRT8* – Non-heamatopoietic cells, *CDH5* and *KDR* – Endothelial cells, *CD34* and *SPN* – YSMP and haematopoietic progenitors, *ITGA2B* and *GYPA* – Megakaryocyte and Erythroid, *PTPRC* and *C1QA* – Macrophage.

Figure S10

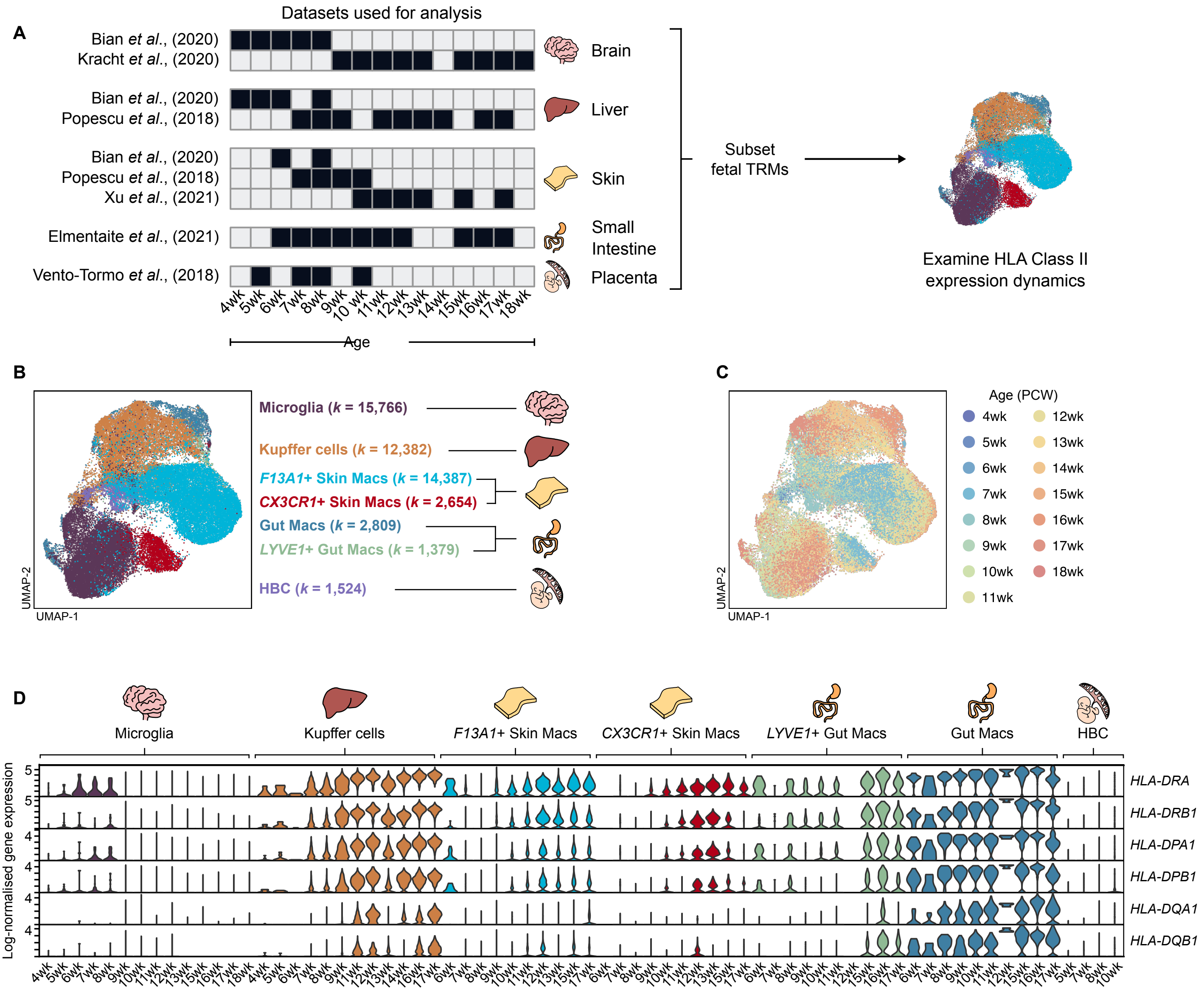

A
Datasets used for analysis
Bian et al., (2020)
Kracht et al., (2020)
Brain
Bian et al., (2020)
Popescu et al., (2018)
Liver
Bian et al., (2020)
Popescu et al., (2018)
Xu et al., (2021)
Skin
Elmentaite et al., (2021)
Small Intestine
Vento-Tormo et al., (2018)
Placenta
4wk 5wk 6wk 7wk 8wk 9wk 10 wk 11wk 12wk 13wk 14wk 15wk 16wk 17wk 18wk
Age
Subset fetal TRMs
Examine HLA Class II expression dynamics
B
Microglia (k = 15,766)
Kupffer cells (k = 12,382)
F13A1+ Skin Macs (k = 14,387)
CX3CR1+ Skin Macs (k = 2,654)
Gut Macs (k = 2,809)
LYVE1+ Gut Macs (k = 1,379)
HBC (k = 1,524)
UMAP-1
UMAP-2
C
Age (PCW)
4wk 5wk 6wk 7wk 8wk 9wk 10wk 11wk 12wk 13wk 14wk 15wk 16wk 17wk 18wk
UMAP-1
UMAP-2
D
Microglia
Kupffer cells
F13A1+ Skin Macs
CX3CR1+ Skin Macs
LYVE1+ Gut Macs
Gut Macs
HBC
Log-normalised gene expression
HLA-DRA
HLA-DRB1
HLA-DPA1
HLA-DPB1
HLA-DQA1
HLA-DQB1

Fig.S10 scRNAseq analysis of human macrophages during foetal **development. A**) Schematic representation of scRNAseq datasets used to construct human foetal macrophage timelines from the brain[24,39], liver[24,25], skin[24,25,37], small intestine[38] and placenta[21]. Black squares denote cells from a given dataset and age were used. Macrophages were subset and batch correction performed, correcting for the study of origin of all cells. **B**) UMAP visualisation of 50,901 human foetal macrophage populations across developmental time, as in Fig5.E, with cells coloured by their identity. Datasets from[21,24,25,37–39]. Number of cells of each identity (*k*) are annotated. **C**) UMAP visualisation with cells coloured by their developmental age in PCW – post-conception weeks. **D**) Log-normalised gene expression violin plots of HLA Class II genes in foetal macrophage populations across developmental time. The genes shown are used to calculate the proportion of cells which are HLA Class II^{pos} in Fig.5E.

Figure S11

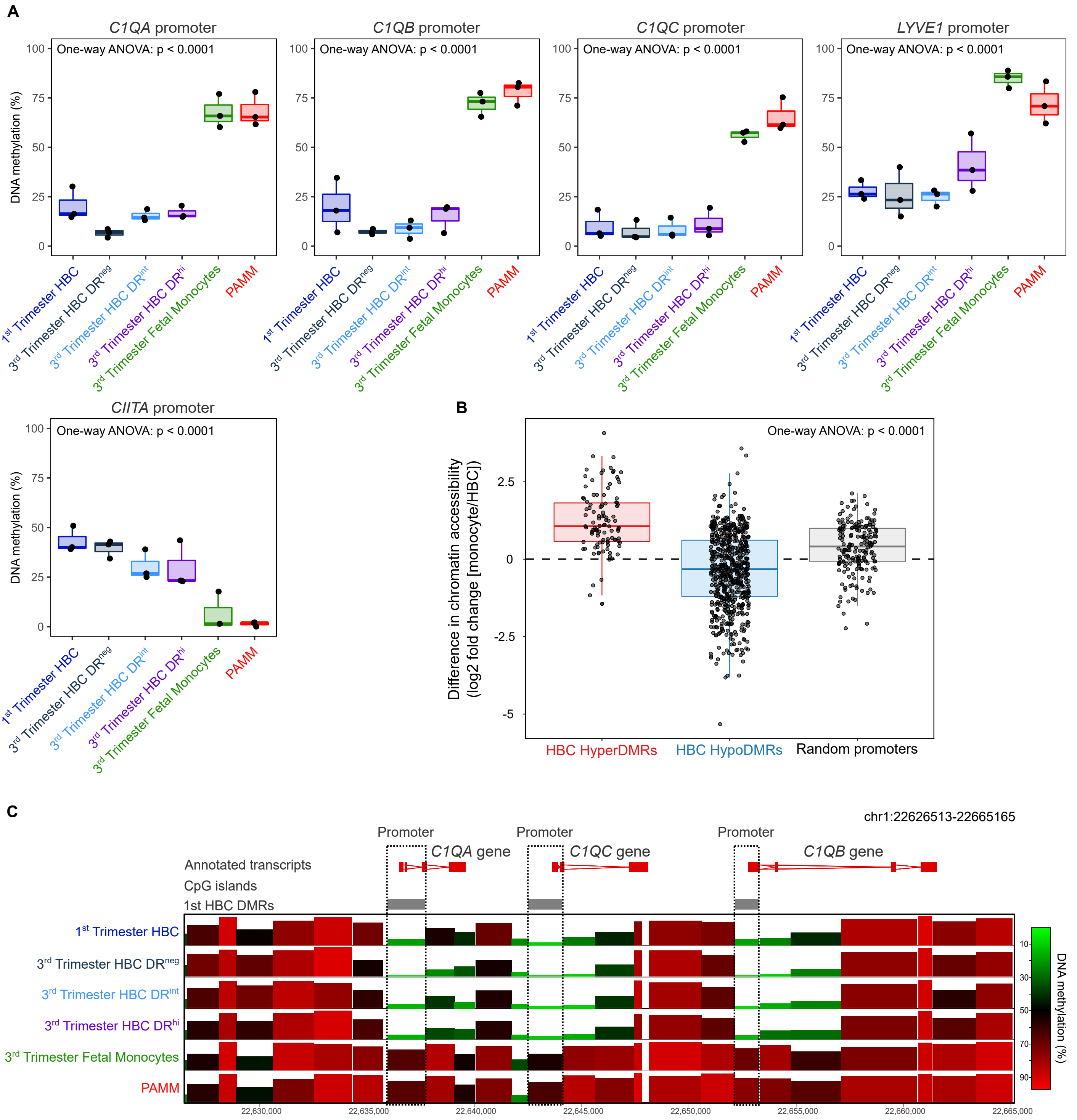
A
C1QA promoter
C1QB promoter
C1QC promoter
LYVE1 promoter
CIITA promoter
One-way ANOVA: p < 0.0001
DNA methylation (%)
100
75
50
25
0
1st Trimester HBC
3rd Trimester HBC DRneg
3rd Trimester HBC DRint
3rd Trimester HBC DRhi
3rd Trimester Fetal Monocytes
PAMM
B
One-way ANOVA: p < 0.0001
Difference in chromatin accessibility (log2 fold change [monocyte/HBC])
2.5
0
-2.5
-5
HBC HyperDMRs
HBC HypoDMRs
Random promoters
C
chr1:22626513-22665165
Promoter
C1QA gene
C1QC gene
C1QB gene
Annotated transcripts
CpG islands
1st HBC DMRs
1st Trimester HBC
3rd Trimester HBC DRneg
3rd Trimester HBC DRint
3rd Trimester HBC DRhi
3rd Trimester Fetal Monocytes
PAMM
22,630,000
22,635,000
22,640,000
22,645,000
22,650,000
22,655,000
22,660,000
22,665,000
DNA methylation (%)
10
30
50
70
90

Fig.S11. Epigenetic profiling of HBC across gestation. A) Boxplots show levels of DNA methylation in first trimester HBCs, third trimester HLA-DR^{neg} (DR^{neg}), HLA-DR^{int} (DR^{int}) and HLA-DR^{hi} (DR^{hi}) HBCs, third trimester foetal monocytes and PAMM cells, for a selection of active (*C1QA*, *C1QC*, *C1QB*, and *LYVE1*) and repressed genes (*CIITA*) in first trimester HBCs (n = 3 donors). Boxplot centre line is the median, with box limits showing the upper and lower quartiles, whiskers as 1.5x interquartile range and overlaid dots show each of the data points. **B)** Boxplot shows average difference in chromatin accessibility between first trimester HBCs and third trimester foetal monocytes at HBC hyper- (k = 106) and hypo-methylated (k = 604) and a random set of promoter 50-CpG windows (k = 186) (ANOVA $p<0.0001$). HyperDMRs show comparatively higher chromatin accessibility (Tukey test, $p<0.01$) concomitant with lower DNA methylation in foetal monocytes, while hypoDMRs show comparatively lower chromatin accessibility (Tukey test, $p<0.01$) concomitant with higher DNA methylation in foetal monocytes. The boxplot centre line is the median, with box limits showing the upper and lower quartiles, whiskers as 1.5x interquartile range and overlaid dots show each of the data points. **C)** Plot shows average DNA methylation for each cell type across the *C1Q* gene cluster. DNA methylation is shown for using 50-CpG windows. The *C1QA*, *C1QC* and *C1QB* gene promoters are highlighted in the dashed boxes.

Supplementary Table 1

REAGENT or RESOURCE	SOURCE	IDENTIFIER	Concentrations			
Antibodies			Aurora Concentration	ARIA III Concentration	Attune Concentration	Concentration IF
Anti-Human CCR2 (PE) (clone K036C2)	BioLegend	Cat#:357205, RRID:AB_2562058	1:40	1:40		
Anti-Human CD10 (BV605) (clone HI10a)	BioLegend	Cat#:312221, RRID:AB_2562156	1:100	1:100		
Anti-Human CD117 (BV605) (clone 104D2)	BioLegend	Cat#:313218, RRID:AB_2562025	1:40			
Anti-Human CD117 (BV785) (clone 104D2)	BioLegend	Cat#:313237, RRID:AB_2629836	1:40	1:40		
Anti-Human CD123 (PerCP/Cy5.5) (clone 6H6)	BioLegend	Cat#:306016, RRID:AB_2264693	1:40	1:40		
Anti-Human CD131 (PE) (clone 1C1)	Thermo Fisher Scientific	Cat#:12-1319-41, RRID:AB_10852856	1:33	1:33		
Anti-Human CD14 (PE/Dazzle) (clone HCD14)	BioLegend	Cat#:325634, RRID:AB_2563625	1:200	1:100	1:100	
Anti-Human CD163 (PE/Cy7) (clone GHI/61)	BioLegend	Cat#:333613, RRID:AB_2562640		1:100	1:100	
Anti-Human CD19 (BUV737) (clone SJ25C1)	BD Biosciences	Cat#:564303, RRID:AB_2716867	1:100			
Anti-Human CD19 (FITC) (clone SJ25C1)	BioLegend	Cat#:363008, RRID:AB_2564171	1:100	1:100		
Anti-Human CD20 (BUV737) (clone 2H7)	BD Biosciences	Cat#:564432, RRID:AB_2687489	1:100			
Anti-Human CD20 (FITC) (clone 2H7)	BioLegend	Cat#:302304, RRID:AB_314252	1:100	1:100		
Anti-Human CD200R (PE) (clone OX-108)	BioLegend	Cat#:329305, RRID:AB_2074201	1:100			
Anti-Human CD235a (APC) (clone HI264)	BioLegend	Cat#:349113, RRID:AB_2650975	1:50			
Anti-Human CD235a (APC/Cy7) (clone HI264)	BioLegend	Cat#:349115, RRID:AB_2650977		1:100	1:100	
Anti-Human CD235a (FITC) (clone HI264)	BioLegend	Cat#:349104, RRID:AB_10613463	1:200	1:100		
Anti-Human CD3 (BUV737) (clone UCHT1)	BD Biosciences	Cat#:564307, RRID:AB_2744390	1:100			
Anti-Human CD3 (FITC) (clone UCHT1)	BioLegend	Cat#:300406, RRID:AB_314060	1:100	1:100		
Anti-Human CD31 (AF488) (clone WM59)	BioLegend	Cat#:303109, RRID:AB_493075				1:50

Anti-Human CD335 (FITC) (clone 9E2)	BioLegend	Cat#:331921, RRID:AB_2561964	1:100	1:100		
Anti-Human CD34 - Mouse (Unconjugated) (clone QBEND-10)	Abcam	Cat#:ab8536, RRID:AB_306607				1:100
Anti-Human CD34 (AF700) (clone 561)	BioLegend	Cat#:343621, RRID:AB_2632722	1:100	1:20		
Anti-Human CD34 (BUV805) (clone 581)	BD Biosciences	Cat#:748388, RRID:AB_2872807	1:100			
Anti-Human CD38 (APC/Cy7) (clone HIT2)	BioLegend	Cat#:303533, RRID:AB_2561604	1:40	1:40		
Anti-Human CD41 (BV421) (clone HIP8)	BioLegend	Cat#:303730, RRID:AB_2629627	1:1000		1:1000	
Anti-Human CD41 (FITC) (clone HIP8)	BioLegend	Cat#:303703, RRID:AB_314373	1:1000	1:1000		
Anti-Human CD41 (PE) (clone HIP8)	BioLegend	Cat#:303705, RRID:AB_314375		1:1000		
Anti-Human CD43 - Rabbit (Unconjugated) (ployclonal)	Atlas Antibodies	Cat#:HPA055244, RRID:AB_2682756				1:100
Anti-Human CD43 (BV480) (clone L60)	BD Biosciences	Cat#:746582, RRID:AB_2743865	1:200			
Anti-Human CD43 (BV605) (clone L60)	BD Biosciences	Cat#:745132, RRID:AB_2742735		1:100		
Anti-Human CD45 - Rat (Unconjugated) (clone YAML501.4)	Thermo Fisher Scientific	Cat#: MA5-17687, RRID:AB_2539077				1:100
Anti-Human CD45 (BUV395) (clone HI30)	BioLegend	Cat#:563792, RRID:AB_2744400	1:50			
Anti-Human CD45 (PerCP/Cy5.5) (clone 2D1)	BioLegend	Cat#:368503, RRID:AB_2566351		1:33	1:33	
Anti-Human CD45RA (PE/Cy7) (clone H100)	BioLegend	Cat#:304125, RRID:AB_10709440	1:100	1:100		
Anti-Human CD56 (BUV737) (clone NCAM16.2)	BD Biosciences	Cat#:564447, RRID:AB_2744432	1:100			
Anti-Human CD56 (FITC) (clone HCD56)	BioLegend	Cat#:318304, RRID:AB_604100	1:100	1:100		
Anti-Human CD66b (AF700) (clone G10F5)	BioLegend	Cat#:305113, RRID:AB_2566037	1:33	1:33	1:33	
Anti-Human CD66b (FITC) (clone G10F5)	BioLegend	Cat#:305103, RRID:AB_314495	1:100	1:100		
Anti-Human CD68 (PE) (clone Y1/82A)	BioLegend	Cat#:333807, RRID:AB_1089057	1:100			
Anti-Human CD71 (PE) (clone CY1G4)	BioLegend	Cat#:334105, RRID:AB_2271603			1:2000	

Anti-Human CD9 (PE/cy7) (clone HI9a)	BioLegend	Cat#:312115, RRID:AB_2728255	1:1000	1:500	1:1000	
Anti-Human CD90 (PE) (clone 5E10)	BioLegend	Cat#:328109, RRID:AB_893442	1:1000			
Anti-Human EGFR (BV605) (clone AY13)	BioLegend	Cat#:352927, RRID:AB_2810558	1:1000			
Anti-Human FCER1A (PE/Cy7) (clone AER-37 (CRA-1))	BioLegend	Cat#:334619, RRID:AB_10588849	1:100			
Anti-Human FOLR2 (APC) (clone 94b/FOLR2)	BioLegend	Cat#:391705, RRID:AB_2721302	1:2000	1:1000	1:1000	
Anti-Human FOLR2 (PE) (clone 94b/FOLR2)	BioLegend	Cat#:391703, RRID:AB_2721335	1:2000	1:1000		
Anti-Human HLA-A2 (AF700) (clone BB7.2)	BioLegend	Cat#:343317, RRID:AB_2561784	1:100	1:100		
Anti-Human HLA-A2 (APC/Cy7) (clone BB7.2)	BioLegend	Cat#:343310, RRID:AB_2561568	1:200	1:100		
Anti-Human HLA-A3 (BV650) (clone GAP.A3)	BD Biosciences	Cat#:747774, RRID:AB_2739760	1:100	1:100		
Anti-Human HLA-DR (BV711) (clone L243)	BioLegend	Cat#:307643, RRID:AB_11218794	1:1000	1:100		
Anti-Human HLA-DR (BV786) (clone G46 - 6)	BD Biosciences	Cat#:564041, RRID:AB_2738559	1:200	1:100	1:100	
Anti-Human HLA-G (FITC) (clone MEM-G/9)	BioRad	Cat#:MCA2044F, RRID:AB_322626	1:100			
Donkey Anti-Mouse IgG Secondary (AF488)	Thermo Fisher Scientific	Cat#:A21202, RRID:AB_141607				1:1000
Donkey Anti-Rabbit IgG Secondary (AF647)	Thermo Fisher Scientific	Cat#:A31573, RRID:AB_2536183				1:1000
Donkey Anti-Rat IgG Secondary (AF594)	Thermo Fisher Scientific	Cat#:A21209, RRID:AB_2535795				1:1000

Supplementary Table 2

REAGENT or RESOURCE	SOURCE	IDENTIFIER
Chemicals, Peptides, and Recombinant Proteins		
4′,6-diamidino-2-phenylindole (DAPI)	Sigma-Aldrich	D9542
Acetone	Sigma-Aldrich	179124-1L
Advanced DMEM/F-12	Thermo Fisher Scientific	12634028
Aqua Zombie Fixable Viability Kit	Biolegend	423101
Collagenase V	Sigma-Aldrich	C9263
DePeX moutning medium 'Gurr'	BDH	361252B
DNAse I	Roche	10104159001
EDTA	Sigma-Aldrich	324506
Eprex epoetin alfa 1,000 IU/0.5ml	Janssen-Cilag	
Fetal Bovine Serum	Sigma-Aldrich	f9665-500ML
Gibco™ HEPES (1M)	Thermo Fischer Scientific	11560496
Giemsa-stain	Sigma-Aldrich	48900-500ML-F
Human AB serum	Sigma-Aldrich	H4522
Human IL-3, premium grade	Miltenyi Biotec	130-095-070
Human IL-6, premium grade	Miltenyi Biotec	130-093-931
Human M-CSF, premium grade	Miltenyi Biotec	130-096-489
Human SCF, premium grade	Miltenyi Biotec	130-096-694
Human TPO, research grade	Miltenyi Biotec	130-095-745
ibidi Mounting Medium	ibidi	50001
L-Glutamine	Sigma-Aldrich	G7513
LIVE/DEAD Fixable Blue Dead Cell Stain Kit	Thermo Fisher Scientific	L23105
Methanol	Thermo Fischer Scientific	10675112
Mouse serum	Sigma-Aldrich	M5905
optimal cutting temperature embedding medium	Thermo Fisher Scientific	12678646
Pancoll	Pan-Biotech	P04-60500
Penicillin Streptomycin	Sigma-Aldrich	P4333
Rat serum	Sigma-Aldrich	R9759-5ML

StemPro-34 SFM (1x) and Nutrient Supplement	Thermo Fisher Scientific	10639011
SUPERase-In RNase Inhibitor (20 U/µl)	Thermo Fisher Scientific	AM2694
Triton X-100	Sigma-Aldrich	X100-500ML
Trypsin	Pan-Biotech	P10-025100P
Software and Algorithms		
biomaRt (R package)		https://bioconductor.org/packages/release/bioc/html/biomaRt.html
Cytofkit2 (R package)		https://github.com/JinmiaoChenLab/cytofkit2
flowCore (R package)		https://www.bioconductor.org/packages/release/bioc/html/flowCore.html
Flowjo v10.6.1	Treestar	https://www.flowjo.com/
ForceAtlas2 (Python alogirthm)		https://github.com/bhargavchippada/forceatlas2
indexSort (R package)		https://github.com/Kawameicha/indexSort
Pheatmap (R package)		https://github.com/raivokolde/pheatmap
Prism 9	Graphpad	https://www.graphpad.com/scientific-software/prism/
R version 3.5.1	The R foundation	https://www.r-project.org/
SCENIC (R package)	Aibar et al., (2017)	https://scenic.aertslab.org/
Seurat v3 (R package)	Butler et al., (2018)	https://satijalab.org/seurat/
SeuratDisk (R package)		https://github.com/mojaveazure/seurat-disk
SeuratWrappers (R package)		https://github.com/satijalab/seurat-wrappers
Publically available scRNAseq data used		
CS10 Embryo Body	Zheng et al., (2019)	GSE135202
CS11 Embryo CH	Zheng et al., (2019)	GSE135202
CS11 YS	Bian et al., (2020)	GSE137010
CS15 AGM	Zheng et al., (2019)	GSE135202
CS7 Embryo and YS	Tyser et al., (2021)	E-MTAB-9388, http://www.human-gastrula.net/
Early TRMs and YSMP	Bian et al., (2020)	GSE133345
Fetal Bone Marrow and Liver	Ranzoni et al., (2021)	E-MTAB-9067, https://gitlab.com/cvejic-group/integrative-scrna-scatac-human-foetal/-/tree/master/Data/ScanpyObjets
Fetal Gut	Elmentaite et al., (2020)	E-MTAB-8901, https://www.gutcellatlas.org/
Fetal Kuppfer Cells	Popescu et al., (2018)	E-MTAB-7407
Fetal Liver all cells	Popescu et al., (2018)	E-MTAB-7407, https://www.covid19cellatlas.org/index.healthy.html
Fetal Microglia	Kracht et al., (2020)	GSE141862
Fetal Skin	Popescu et al., (2018)	E-MTAB-7407
Fetal Skin	Xu et al., (2021)	GSE179565

HBC	Vento-Tormo et al., (2018)	E-MTAB-6701
Murine AGM, FL and YS	Zhu et al., (2020)	GSE137116
Murine Placenta	Liang et al., (2021)	GSE152903

Supplementary Table 3

ID	Cell type	Cell number	Sample ID	Trimester	Raw Reads	Unique align	Duplication	CpGme	CHHme
Y042	1st HBC DRneg	100.000	1st HBC rep1	1	88.159.704	44,7%	10,0%	65,6%	1,2%
Y034	1st HBC DRneg	20.000	1st HBC rep2	1	78.148.664	37,1%	9,9%	68,0%	1,1%
Y017	1st HBC DRneg	5.000	1st HBC rep3	1	32.146.086	38,5%	11,8%	67,4%	1,0%
FT7282	3rd HBC DRneg	20.000	3rd HBC DRneg rep1	3	40.907.407	38,3%	10,5%	66,2%	1,4%
FT2711	3rd HBC DRneg	20.000	3rd HBC DRneg rep2	3	66.641.535	39,0%	13,2%	64,8%	1,0%
FT4702	3rd HBC DRneg	20.000	3rd HBC DRneg rep3	3	59.266.939	40,9%	9,8%	65,9%	0,9%
FT7282	HBC DRint	20.000	3rd HBC DRint rep1	3	39.175.538	39,5%	10,2%	67,5%	1,4%
FT2711	HBC DRint	20.000	3rd HBC DRint rep2	3	50.953.966	40,6%	14,3%	68,1%	1,1%
FT4702	HBC DRint	20.000	3rd HBC DRint rep3	3	57.204.076	39,8%	11,3%	68,2%	1,1%
FT7282	HBC DRhigh	20.000	3rd HBC DRhi rep1	3	60.988.994	41,3%	9,8%	67,2%	1,2%
FT2711	HBC DRhigh	20.000	3rd HBC DRhi rep2	3	79.077.243	39,1%	13,0%	69,4%	1,2%
FT4702	HBC DRhigh	5.800	3rd HBC DRhi rep3	3	68.667.518	41,3%	11,8%	69,7%	1,2%
FT7282	3rd Fetal mono	20.000	Foetal mono rep1	3	39.514.876	38,1%	10,5%	73,0%	1,0%
FT2711	3rd Fetal mono	20.000	Foetal mono rep2	3	45.414.728	39,1%	12,4%	72,5%	1,0%
FT4702	3rd Fetal mono	20.000	Foetal mono rep3	3	52.578.987	43,3%	15,1%	72,8%	1,0%
Y042	PAMM1	30.000	PAMM1 rep1	1	85.244.882	37,0%	9,8%	72,4%	1,3%
FT7282	PAMM1	20.000	PAMM1 rep2	3	52.232.569	39,4%	11,0%	73,4%	1,0%
FT2711	PAMM1	20.000	PAMM1 rep3	3	44.090.580	42,1%	11,1%	71,8%	1,2%
