# Supplementary material for: Primitive haematopoiesis in the human placenta gives rise to macrophages with epigenetically silenced HLA-DR
Source: Nat Commun. 2023 Mar 30;14:1764. doi: 10.1038/s41467-023-37383-2 (PMC10063560; doi:10.1038/s41467-023-37383-2)
Supplement: Supplementary file 2 — Reporting Summary [file 41467_2023_37383_MOESM2_ESM.pdf]

nature portfolio

Corresponding author(s): Courtney Hanna and Naomi McGovern

Last updated by author(s): Mar 6, 2023

Reporting Summary

Nature Portfolio wishes to improve the reproducibility of the work that we publish. This form provides structure for consistency and transparency in reporting. For further information on Nature Portfolio policies, see our Editorial Policies and the Editorial Policy Checklist.

Statistics

For all statistical analyses, confirm that the following items are present in the figure legend, table legend, main text, or Methods section.

n/a	Confirmed	
☐	☒	The exact sample size (n) for each experimental group/condition, given as a discrete number and unit of measurement
☐	☒	A statement on whether measurements were taken from distinct samples or whether the same sample was measured repeatedly
☐	☒	The statistical test(s) used AND whether they are one- or two-sided<br>*Only common tests should be described solely by name; describe more complex techniques in the Methods section.*
☒	☐	A description of all covariates tested
☐	☒	A description of any assumptions or corrections, such as tests of normality and adjustment for multiple comparisons
☐	☒	A full description of the statistical parameters including central tendency (e.g. means) or other basic estimates (e.g. regression coefficient) AND variation (e.g. standard deviation) or associated estimates of uncertainty (e.g. confidence intervals)
☐	☒	For null hypothesis testing, the test statistic (e.g. F, t, r) with confidence intervals, effect sizes, degrees of freedom and P value noted<br>*Give P values as exact values whenever suitable.*
☒	☐	For Bayesian analysis, information on the choice of priors and Markov chain Monte Carlo settings
☒	☐	For hierarchical and complex designs, identification of the appropriate level for tests and full reporting of outcomes
☒	☐	Estimates of effect sizes (e.g. Cohen's d, Pearson's r), indicating how they were calculated

Our web collection on statistics for biologists contains articles on many of the points above.

Software and code

Policy information about availability of computer code

Data collection	Software used include: R, Python 3, BD FACS DIVA software, flowjo, Axiovision and ZEN software (zeiss), Prism 9 Graphpad Photoshop, Illustrator, Illumina Next Generation Sequencing Platforms, Trim Galore v0.6.6, Bismark v0.16.3.<br><br>Detailed parameters of each of the methods are mentioned in relevant sections in Methods.
Data analysis	Software used include: R v3.6.3 (The R foundation) , Python 3, BD FACS DIVA software,, Axiovision and ZEN software (zeiss),Prism 9 Graphpad, Flowjo v10.7, Photoshop, Illustrator, SeqMonk, RStudio.<br><br>Detailed parameters of each of the methods are mentioned in relevant sections in Methods.<br><br>Data analysis was performed using:<br>R v3.6.3 (The R foundation)<br>Trim Galore v0.6.6: https://github.com/FelixKrueger/TrimGalore<br>STAR v2.5.0.a: https://github.com/alexdobin/STAR<br>biomaRt (R package): https://bioconductor.org/packages/release/bioc/html/biomaRt.html<br>Cytofkit2 (R package): https://github.com/JinmiaoChenLab/cytofkit2<br>batchelor (R package): https://bioconductor.org/packages/release/bioc/html/batchelor.html<br>flowCore (R package): https://www.bioconductor.org/packages/release/bioc/html/flowCore.html<br>Flowjo v10.7 Treestar: https://www.flowjo.com/

ForceAtlas2 (Python alogirthm): https://github.com/bhargavchippada/forceatlas2
indexSort (R package): https://github.com/Kawameicha/indexSort
Pheatmap (R package): https://github.com/raivokolde/pheatmap
Prism 9 Graphpad: https://www.graphpad.com/scientific-software/prism/
R version 3.5.1 The R foundation: https://www.r-project.org/
SeqMonk v1.48.0: https://www.bioinformatics.babraham.ac.uk/projects/seqmonk/
ggplot2 (R package): https://ggplot2.tidyverse.org/
SCENIC (R package), Aibar et al., (2017): https://scenic.aertslab.org/
Seurat v3 (R package), Butler et al., (2018): https://satijalab.org/seurat/
SeuratDisk (R package): https://github.com/mojaveazure/seurat-disk
SeuratWrappers (R package): https://github.com/satijalab/seurat-wrappers

For manuscripts utilizing custom algorithms or software that are central to the research but not yet described in published literature, software must be made available to editors and reviewers. We strongly encourage code deposition in a community repository (e.g. GitHub). See the Nature Portfolio guidelines for submitting code & software for further information.

Data

Policy information about availability of data

All manuscripts must include a data availability statement. This statement should provide the following information, where applicable:

- Accession codes, unique identifiers, or web links for publicly available datasets
- A description of any restrictions on data availability
- For clinical datasets or third party data, please ensure that the statement adheres to our policy

The following publically available datasets were used in this study:
CS10 Embryo Body Zheng et al., (2019) GSE135202
CS11 Embryo CH Zheng et al., (2019) GSE135202
CS11 YS Bian et al., (2020) GSE137010
CS15 AGM Zheng et al., (2019) GSE135202
CS7 Embryo and YS Tyser et al., (2021) E-MTAB-9388, http://www.human-gastrula.net/
Early TRMs and YSMP Bian et al., (2020) GSE133345
Fetal Bone Marrow and Liver Ranzoni et al., (2021) E-MTAB-9067, https://gitlab.com/cvejic-group/integrative-scrna-scatac-human-foetal/-/tree/master/Data/ScanpyObjets
Fetal Gut Elmentaite et al., (2020) E-MTAB-8901, https://www.gutcellatlas.org/
Fetal Kuppfer Cells Popescu et al., (2018) E-MTAB-7407
Fetal Liver all cells Popescu et al., (2018) E-MTAB-7407, https://www.covid19cellatlas.org/index.healthy.html
Fetal Microglia Kracht et al., (2020) GSE141862
Fetal Skin Popescu et al., (2018) E-MTAB-7407
Fetal Skin Xu et al., (2021) GSE179565
HBC Vento-Tormo et al., (2018) E-MTAB-6701
Murine AGM, FL and YS Zhu et al., (2020) GSE137116
Murine Placenta Liang et al., (2021) GSE152903
Murine placental data was obtained from GSE152903 and murine FL, AGM and YS data was obtained from GSE137116.

Human research participants

Policy information about studies involving human research participants and Sex and Gender in Research.

Reporting on sex and gender	For our single cell RNA sequencing, we used sex to distinguish fetal (male) from maternal cells. For all other experiments samples were not segregated based on sex, samples are both male and female.
Population characteristics	Organ donors were either male or female. Ethnicity was not recorded, but expected to be primarily Caucasian
Recruitment	Maternal consent was obtained through Cambridge Addenbrooke's hospital by a Research Nurse. First trimester placental tissues were obtained from healthy women with apparently normal pregnancies undergoing elective first trimester terminations (6-12 weeks estimated gestational age (EGA)). Term placental samples were obtained from healthy women undergoing elective cesarean sections. Peripheral blood was taken from healthy adult volunteers. All samples were obtained with written informed consent from participants under ethical approval which was obtained from the Cambridge Research Ethics committee (study 04/Q0108/23).
Ethics oversight	Cambridge Research Ethics committee (study 04/Q0108/23).

Note that full information on the approval of the study protocol must also be provided in the manuscript.

Field-specific reporting

Please select the one below that is the best fit for your research. If you are not sure, read the appropriate sections before making your selection.

- [x] Life sciences
- [ ] Behavioural & social sciences
- [ ] Ecological, evolutionary & environmental sciences

For a reference copy of the document with all sections, see nature.com/documents/nr-reporting-summary-flat.pdf

Life sciences study design

All studies must disclose on these points even when the disclosure is negative.

Sample size	No statistical methods were used to predetermine sample size. We followed standards in the field.
Data exclusions	No exclusion was applied to the uploaded raw data in ArrayExpress. For the final count matrix, we excluded cells based on pre-established criteria for single-cells: we excluded low quality samples and contaminating cells (i.e. - cells with low number of detected genes and high mitochondria content) - exclusion criteria for each case are comprehensively detailed in the relevant Methods section.
Replication	scRNAseq was carried our on 2 placentas from 6-14 gestational weeks. Low-input bisulphite sequencing was carried out on sorted cell types isolated from 3 first trimester and 3 term placentas. Additional samples were used for the validation experiments and the sample size for each experiment is provided in the Figure legends. Any replication experiment was excluded.
Randomization	Only healthy individuals were considered in our analysis. Therefore, any further randomization protocol was required.
Blinding	Only healthy individuals were considered in our analysis. Therefore, no blinding was performed

Reporting for specific materials, systems and methods

We require information from authors about some types of materials, experimental systems and methods used in many studies. Here, indicate whether each material, system or method listed is relevant to your study. If you are not sure if a list item applies to your research, read the appropriate section before selecting a response.

Materials & experimental systems

n/a	Involved in the study	
	x	Antibodies
x		Eukaryotic cell lines
x		Palaeontology and archaeology
x		Animals and other organisms
x		Clinical data
x		Dual use research of concern

Methods

n/a	Involved in the study	
x		ChIP-seq
	x	Flow cytometry
x		MRI-based neuroimaging

Antibodies

Antibodies used

Anti-Human CCR2 (PE) (clone K036C2)BioLegen Cat#:357205, RRID:AB_2562058; Anti-Human CD10 (BV605) (clone HI10a) BioLegend Cat#:312221, RRID:AB_2562156; Anti-Human CD117 (BV605) (clone 104D2)BioLegend Cat#:313218, RRID:AB_2562025; Anti-Human CD117 (BV785) (clone 104D2) BioLegen Cat#:313237, RRID:AB_2629836; Anti-Human CD123 (PerCP/Cy5.5) (clone 6H6) BioLegend Cat#:306016, RRID:AB_2264693; Anti-Human CD131 (PE) (clone 1C1) Thermo Fisher Scientific Cat#:12-1319-41, RRID:AB_10852856; Anti-Human CD14 (PE/Dazzle) (clone HCD14) BioLegend Cat#:325634, RRID:AB_2563625; Anti-Human CD163 (PE/Cy7) (clone GHI/61) BioLegend Cat#:333613, RRID:AB_2562640; Anti-Human CD19 (BUV737) (clone SJ25C1) BD Biosciences Cat#:564303, RRID:AB_2716867; Anti-Human CD19 (FITC) (clone SJ25C1) BioLegend Cat#:363008, RRID:AB_2564171; Anti-Human CD20 (BUV737) (clone 2H7) BD Biosciences Cat#:564432, RRID:AB_2687489; Anti-Human CD20 (FITC) (clone 2H7) BioLegend Cat#:302304, RRID:AB_314252; Anti-Human CD200R (PE) (clone OX-108) BioLegend Cat#:329305, RRID:AB_2074201; Anti-Human CD235a (APC) (clone HI264) BioLegend Cat#:349113, RRID:AB_2650975; Anti-Human CD235a (APC/Cy7) (clone HI264) BioLegend Cat#:349115, RRID:AB_2650977; Anti-Human CD235a (FITC) (clone HI264) BioLegend Cat#:349104, RRID:AB_10613463; Anti-Human CD3 (BUV737) (clone UCHT1) BD Biosciences Cat#:564307, RRID:AB_2744390; Anti-Human CD3 (FITC) (clone UCHT1) BioLegend Cat#:300406, RRID:AB_314060; Anti-Human CD31 (AF488) (clone WM59) BioLegend Cat#:303109, RRID:AB_493075; Anti-Human CD335 (FITC) (clone 9E2) BioLegend Cat#:331921, RRID:AB_2561964; Anti-Human CD34 - Mouse (Unconjugated) (clone QBEND-10) Abcam Cat#:ab8536, RRID:AB_306607; Anti-Human CD34 (AF700) (clone 561) BioLegend Cat#:343621, RRID:AB_2632722; Anti-Human CD34 (BUV805) (clone 581) BD Biosciences Cat#:748388, RRID:AB_2872807; Anti-Human CD38 (APC/Cy7) (clone HIT2) BioLegend Cat#:303533, RRID:AB_2561604; Anti-Human CD41 (BV421) (clone HIP8) BioLegend Cat#:303730, RRID:AB_2629627; Anti-Human CD41 (FITC) (clone HIP8) BioLegend Cat#:303703, RRID:AB_314373; Anti-Human CD41 (PE) (clone HIP8) BioLegend Cat#:303705, RRID:AB_314375; Anti-Human CD43 - Rabbit (Unconjugated) (ploygonal) Atlas Antibodies Cat#:HPA055244, RRID:AB_2682756; Anti-Human CD43 (BV480) (clone L60) BD Biosciences Cat#:746582, RRID:AB_2743865; Anti-Human CD43 (BV605) (clone L60) BD Biosciences Cat#:745132, RRID:AB_2742735; Anti-Human CD45 - Rat (Unconjugated) (clone YAML501.4) Thermo Fisher Scientific

Cat#: MA5-17687, RRID:AB_2539077; Anti-Human CD45 (BUV395) (clone HI30) BioLegend Cat#:563792, RRID:AB_2744400; Anti-Human CD45 (PerCP/Cy5.5) (clone 2D1) BioLegend Cat#:368503, RRID:AB_2566351; Anti-Human CD45RA (PE/Cy7) (clone H100) BioLegend Cat#:304125, RRID:AB_10709440; Anti-Human CD56 (BUV737) (clone NCAM16.2) BD Biosciences Cat#:564447, RRID:AB_2744432; Anti-Human CD56 (FITC) (clone HCD56) BioLegend Cat#:318304, RRID:AB_604100; Anti-Human CD66b (AF700) (clone G10F5) BioLegend Cat#:305113, RRID:AB_2566037; Anti-Human CD66b (FITC) (clone G10F5) BioLegend Cat#:305103, RRID:AB_314495; Anti-Human CD68 (PE) (clone Y1/82A) BioLegend Cat#:333807, RRID:AB_1089057; Anti-Human CD71 (PE) (clone CY1G4) BioLegend Cat#:334105, RRID:AB_2271603; Anti-Human CD9 (PE/cy7) (clone HI9a) BioLegend Cat#:312115, RRID:AB_2728255; Anti-Human CD90 (PE) (clone 5E10) BioLegend Cat#:328109, RRID:AB_893442;Anti-Human EGFR (BV605) (clone AY13) BioLegend Cat#:352927, RRID:AB_2810558; Anti-Human FCER1A (PE/Cy7) (clone AER-37 (CRA-1)) BioLegend Cat#:334619, RRID:AB_10588849; Anti-Human FOLR2 (APC) (clone 94b/FOLR2) BioLegend Cat#:391705, RRID:AB_2721302; Anti-Human FOLR2 (PE) (clone 94b/FOLR2) BioLegend Cat#:391703, RRID:AB_2721335; Anti-Human HLA-A2 (AF700) (clone BB7.2) BioLegend Cat#:343317, RRID:AB_2561784; Anti-Human HLA-A2 (APC/Cy7) (clone BB7.2) BioLegend Cat#:343310, RRID:AB_2561568; Anti-Human HLA-A3 (BV650) (clone GAP.A3) BD Biosciences Cat#:747774, RRID:AB_2739760; Anti-Human HLA-DR (BV711) (clone L243) BioLegend Cat#:307643, RRID:AB_11218794; Anti-Human HLA-DR (BV786) (clone G46 - 6) BD Biosciences Cat#:564041, RRID:AB_2738559; Anti-Human HLA-G (FITC) (clone MEM-G/9) BioRad Cat#:MCA2044F, RRID:AB_322626; Donkey Anti-Mouse IgG Secondary (AF488) Thermo Fisher Scientific Cat#:A21202, RRID:AB_141607; Donkey Anti-Rabbit IgG Secondary (AF647) Thermo Fisher Scientific Cat#:A31573, RRID:AB_2536183; Donkey Anti-Rat IgG Secondary (AF594) Thermo Fisher Scientific Cat#:A21209, RRID:AB_2535795

Validation

Anti-Human CCR2 (PE) (clone K036C2) BioLegend Cat#:357205, RRID:AB_2562058 Staining of human peripheral blood monocytes with CD192 (clone K036C2) PE. FACS plot shown on website.
Anti-Human CD10 (BV605) (clone HI10a) BioLegend Cat#:312221, RRID:AB_2562156 Staining of human peripheral blood granulocytes with anti-human CD10 (clone HI10a) Brilliant Violet 605™. FACS plot shown on website.
Anti-Human CD117 (BV605) (clone 104D2) BioLegend Cat#:313218, RRID:AB_2562025 Staining ofhuman erythroleukemia cell line (HEL) with CD117 (clone 104D2) Brilliant Violet 605™. FACS plot shown on website.
Anti-Human CD117 (BV785) (clone 104D2) BioLegend Cat#:313237, RRID:AB_2629836 Staining of human erythroleukemia cell line (HEL) with CD117 (clone 104D2) Brilliant Violet 785™. FACS plot shown on website.
Anti-Human CD123 (PerCP/Cy5.5) (clone 6H6) BioLegend Cat#:306016, RRID:AB_2264693 Staining of human peripheral blood lymphocytes with 6H6 PerCP/Cyanine5.5. FACS plot shown on website.
Anti-Human CD131 (PE) (clone 1C1) Thermo Fisher Scientific Cat#:12-1319-41, RRID:AB_10852856 Staining of normal human peripheral blood cells with Anti-Human CD131 PE (right). FACS plot shown on website.
Anti-Human CD14 (PE/Dazzle) (clone HCD14) BioLegend Cat#:325634, RRID:AB_2563625 Staining of human peripheral blood monocytes with CD14 (clone HCD14) PE/Dazzle™ 594. FACS plot shown on website.
Anti-Human CD163 (PE/Cy7) (clone GHI/61) BioLegend Cat#:333613, RRID:AB_2562640 Staining of human peripheral blood monocytes with CD163 (clone GHI/63) PE/Cyanine7. FACS plot shown on website.
Anti-Human CD19 (BUV737) (clone SJ25C1) BD Biosciences Cat#:564303, RRID:AB_2716867 Staining of human peripheral blood lymphocytes with anti-Human CD19 (BUV737) (clone SJ25C1). FACS plot shown on website.
Anti-Human CD19 (FITC) (clone SJ25C1) BioLegend Cat#:363008, RRID:AB_2564171 Staining of human peripheral blood lymphocytes with CD19 (clone SJ25C1) FITC. FACS plot shown on website.
Anti-Human CD20 (BUV737) (clone 2H7) BD Biosciences Cat#:564432, RRID:AB_2687489 Staining of human peripheral blood lymphocytes with anti-Human CD20 (BUV737) (clone 2H7). FACS plot shown on website.
Anti-Human CD20 (FITC) (clone 2H7) BioLegend Cat#:302304, RRID:AB_314252 Staining of human peripheral blood lymphocytes with anti-CD20 (clone 2H7) FITC. FACS plot shown on website.
Anti-Human CD200R (PE) (clone OX-108) BioLegend Cat#:329305, RRID:AB_2074201 Staining of human peripheral blood lymphocytes with CD3 (UCHT1) APC. FACS plot shown on website.
Anti-Human CD235a (APC) (clone HI264) BioLegend Cat#:349113, RRID:AB_2650975 Staining of human red blood cells with anti-human CD235a (clone HI264) APC. FACS plot shown on website.
Anti-Human CD235a (APC/Cy7) (clone HI264) BioLegend Cat#:349115, RRID:AB_2650977 Staining of human red blood cells with anti-human CD235a (clone HI264) APC/Cyanine7. FACS plot shown on website.
Anti-Human CD235a (FITC) (clone HI264) BioLegend Cat#:349104, RRID:AB_10613463 Staining of human red blood cells stained with HI264 FITC. FACS plot shown on website.
Anti-Human CD3 (BUV737) (clone UCHT1) BD Biosciences Cat#:564307, RRID:AB_2744390 Staining of human peripheral blood lymphocytes with anti-Human CD3 (BUV737) (clone UCHT1). FACS plot shown on website.
Anti-Human CD3 (FITC) (clone UCHT1) BioLegend Cat#:300406, RRID:AB_314060 Staining of human peripheral blood lymphocytes stained with UCHT1 FITC. FACS plot shown on website.
Anti-Human CD31 (AF488) (clone WM59) BioLegend Cat#:303109, RRID:AB_493075 Staining of human peripheral blood granulocytes with CD31 (clone WM59) Alexa Fluor® 488. FACS plot shown on website.
Anti-Human CD335 (FITC) (clone 9E2) BioLegend Cat#:331921, RRID:AB_2561964 Staining of human peripheral blood lymphocytes with CD335 (clone 9E2) FITC. FACS plot shown on website.
Anti-Human CD34 - Mouse (Unconjugated) (clone QBEND-10) Abcam Cat#:ab8536, RRID:AB_306607 Staining of Jurkat cells with ab8536. FACS plot shown on website. Immunohistochemistry analysis of Human colon tissue sections labelling CD34 with ab8536. Blood vessels show positive staining. Image provided.
Anti-Human CD34 (AF700) (clone 561) BioLegend Cat#:343621, RRID:AB_2632722 Staining of human peripheral blood mononuclear cells with CD34 (clone 561) Alexa Fluor® 700. FACS plot shown on website.
Anti-Human CD34 (BUV805) (clone 581) BD Biosciences Cat#:748388, RRID:AB_2872807 Manufactures state antibody underwent strigent testing, however FACS plot is not provided. FACS plot is provided for human peripheral blood stem cells stained with this antibody clone but conjugated with other fluorophores such as APC.
Anti-Human CD38 (APC/Cy7) (clone HIT2) BioLegend Cat#:303533, RRID:AB_2561604 Staining of human peripheral blood lymphocytes with CD38 (clone HIT2) APC/Cyanine7. FACS plot shown on website.
Anti-Human CD41 (BV421) (clone HIP8) BioLegend Cat#:303730, RRID:AB_2629627 Staining of human peripheral blood platelets with CD41 (clone HIP8) Brilliant Violet 421™. FACS plot shown on website.
Anti-Human CD41 (FITC) (clone HIP8) BioLegend Cat#:303703, RRID:AB_314373 Staining of human peripheral blood plateletswith HIP8 FITC. FACS plot shown on website.

Anti-Human CD41 (PE) (clone HIP8) BioLegend Cat#:303705, RRID:AB_314375 Staining of human platelets stained with HIP8 PE. FACS plot shown on website.
Anti-Human CD43 - Rabbit (Unconjugated) (ployclonal) Atlas Antibodies Cat#:HPA055244, RRID:AB_2682756 Validated by Human Protein Cell Atlas Project.
Anti-Human CD43 (BV480) (clone L60) BD Biosciences Cat#:746582, RRID:AB_2743865 Manufactures state antibody underwent strigent testing, however FACS plot is not provided. FACS plot is provided for human peripheral blood cells stained with this antibody clone but conjugated with other fluorophore.
Anti-Human CD43 (BV605) (clone L60) BD Biosciences Cat#:745132, RRID:AB_2742735 Manufactures state antibody underwent strigent testing, however FACS plot is not provided. FACS plot is provided for human peripheral blood cells stained with this antibody clone but conjugated with other fluorophore.
Anti-Human CD45 - Rat (Unconjugated) (clone YAML501.4) Thermo Fisher Scientific Cat#: MA5-17687, RRID:AB_2539077 Staining of human peripheral blood monocytes with anti-human CD45 (MA5-17687). FACS plots and microscopy image shown on website.
Anti-Human CD45 (BUV395) (clone HI30) BD Biosciences Cat#:563792, RRID:AB_2744400 Staining of human peripheral blood lymphocytes with anti-Human CD45 (BUV395) (clone HI30). FACS plot shown on website.
Anti-Human CD45 (PerCP/Cy5.5) (clone 2D1) BioLegend Cat#:368503, RRID:AB_2566351 Staining of human peripheral blood lymphocytes with CD45 (clone 2D1) PerCP/Cyanine5.5. FACS plot shown on website.
Anti-Human CD45RA (PE/Cy7) (clone H100) BioLegend Cat#:304125, RRID:AB_10709440 Staining of human peripheral blood lymphocytes with HI100 PE/Cyanine7. FACS plot shown on website.
Anti-Human CD56 (BUV737) (clone NCAM16.2) BD Biosciences Cat#:564447, RRID:AB_2744432 Staining of human peripheral blood lymphocytes with Anti-Human CD56 (BUV737) (clone NCAM16.2). FACS plot shown on website.
Anti-Human CD56 (FITC) (clone HCD56) BioLegend Cat#:318304, RRID:AB_604100 Staining of human peripheral blood lymphocytes with HCD56 FITC. FACS plot shown on website.
Anti-Human CD66b (AF700) (clone G10F5) BioLegend Cat#:305113, RRID:AB_2566037 Staining of human peripheral blood granulocytes with CD66b (clone G10F5) Alexa Fluor® 700. FACS plot shown on website.
Anti-Human CD66b (FITC) (clone G10F5) BioLegend Cat#:305103, RRID:AB_314495 Staining of human peripheral whole blood granulocytes with G10F5 FITC. FACS plot shown on website.
Anti-Human CD68 (PE) (clone Y1/82A) BioLegend Cat#:333807, RRID:AB_1089057 Staining of human peripheral blood monocytes intracellularly with Y1/82A PE. FACS plot shown on website.
Anti-Human CD71 (PE) (clone CY1G4) BioLegend Cat#:334105, RRID:AB_2271603 Staining of PHA-stimulated (3 days) human peripheral blood lymphocytes with CY1G4 PE. FACS plot shown on website.
Anti-Human CD9 (PE/cy7) (clone HI9a) BioLegend Cat#:312115, RRID:AB_2728255 Staining of human platelets with CD9 (clone HI9a) PE/Cyanine7. FACS plot shown on website.
Anti-Human CD90 (PE) (clone 5E10) BioLegend Cat#:328109, RRID:AB_893442 Staining of human erythroleukemic cell line HEL with 5E10 PE . FACS plot shown on website.
Anti-Human EGFR (BV605) (clone AY13) BioLegend Cat#:352927, RRID:AB_2810558 Staining human cervical cancer cell line HELA with EGFR (clone AY13) Brilliant Violet 605™. FACS plot shown on website.
Anti-Human FCER1A (PE/Cy7) (clone AER-37 (CRA-1)) BioLegend Cat#:334619, RRID:AB_10588849 Staining of human peripheral blood leukocytes with AER-37 PE/Cyanine7. FACS plot shown on website.
Anti-Human FOLR2 (APC) (clone 94b/FOLR2) BioLegend Cat#:391705, RRID:AB_2721302 Staining of human peripheral blood monocytes with anti-human FR-β (94b/FOLR2) APC. FACS plot shown on website.
Anti-Human FOLR2 (PE) (clone 94b/FOLR2) BioLegend Cat#:391703, RRID:AB_2721335 Staining of human peripheral blood monocytes with anti-human FR-β (clone 94b/FOLR2) PE. FACS plot shown on website.
Anti-Human HLA-A2 (AF700) (clone BB7.2) BioLegend Cat#:343317, RRID:AB_2561784 Staining of human peripheral blood lymphocytes from HLA-A2 positive donors with anti-human HLA-A2 (clone BB7.2) Alexa Fluor® 700. FACS plot shown on website.
Anti-Human HLA-A2 (APC/Cy7) (clone BB7.2) BioLegend Cat#:343310, RRID:AB_2561568 Staining of human peripheral blood lymphocytes from HLA-A2 positive donors with anti-human HLA-A2 (clone BB7.2) APC/Cyanine7. FACS plot shown on website.
Anti-Human HLA-A3 (BV650) (clone GAP.A3) BD Biosciences Cat#:747774, RRID:AB_2739760 Manufactuere verified antibody in-house on humans, but data is not provided.=. We verified the antibody by FACS in-house on HLA-A3 positive donors (confirmed by PCR).
Anti-Human HLA-DR (BV711) (clone L243) BioLegend Cat#:307643, RRID:AB_11218794 Staining of human peripheral blood lymphocytes with HLA-DR (clone L243) Brilliant Violet 711™. FACS plot shown on website.
Anti-Human HLA-DR (BV786) (clone G46 - 6) BD Biosciences Cat#:564041, RRID:AB_2738559 Staining of human peripheral blood lymphocytes with Anti-Human HLA-DR (BV786) (clone G46 - 6) BD Horizon™ BV786. FACS plot shown on website.
Anti-Human HLA-G (FITC) (clone MEM-G/9) BioRad Cat#:MCA2044F, RRID:AB_322626 Published image is provided on manufacturers website. J Clin Invest. 2010 Nov 1; 120(11): 4102–4110.
Donkey Anti-Mouse IgG Secondary (AF488) Thermo Fisher Scientific Cat#:A21202, RRID:AB_141607 Immunofluorescent microscopy images are provided of HuC/D cells were stained with anti-HuC/D antibody (Product # A-21271) and then incubated with Alexa Fluor 488 conjugated donkey anti-mouse (Product # A-21202) antibody at a dilution of 1:1000 for 30 min. Images provided were taken on a Thermo Fisher Scientific EVOS M5000 Cell Imaging System.
Donkey Anti-Rabbit IgG Secondary (AF647) Thermo Fisher Scientific Cat#:A31573, RRID:AB_2536183 Immunofluorescent microscopy images are provided of human iPSC-derived forebrain organoids derived at Day 40. Cells were stained with a rabbit SOX2 polyclonal antibody (Product # PA1-094X) and Donkey anti-Rabbit Alexa Fluor 647 (Product # A-31573). Images were taken at 20X magnification.
Donkey Anti-Rat IgG Secondary (AF594) Thermo Fisher Scientific Cat#:A21209, RRID:AB_2535795 Immunofluorescent microscopy images are provided of donkey anti-Rat IgG (H+L) Secondary Antibody, Alexa Fluor 594 conjugate was performed using A549 cells stained with alpha Tubulin (YL1/2) Rat Monoclonal Antibody (Product # MA1-80017). The images were captured at 60X magnification.

Flow Cytometry

Plots

Confirm that:

- [x] The axis labels state the marker and fluorochrome used (e.g. CD4-FITC).
- [x] The axis scales are clearly visible. Include numbers along axes only for bottom left plot of group (a 'group' is an analysis of identical markers).
- [x] All plots are contour plots with outliers or pseudocolor plots.
- [x] A numerical value for number of cells or percentage (with statistics) is provided.

Methodology

Sample preparation	Samples were processed immediately upon receipt as previously reported. Samples were washed in PBS for 10 minutes with a stirrer before processing. The placental villi were scraped from the chorionic membrane with a scalpel and digested with 0.2% Trypsin (Pan-Biotech)/ 0.02% Ethylenediaminetetraacetic acid (EDTA) (Source BioScience) at 37oC with stirring, for 7 minutes (first trimester samples) or 10 minutes (term samples). The digested cell suspension was passed through a sterile muslin gauze, and fetal bovine serum (FBS) (Sigma-aldrich) was added to halt the digestion process. The undigested tissue left on the gauze was scraped off with a scalpel and digested in 2.5ml 1mg/ml collagenase V (Sigma-Aldrich), supplemented with 50microlitre of 10mg/ml DNAse I (Roche) for 20 minutes (first trimester samples) or 45 minutes (term samples) at 37oC with agitation. The digested cell suspension was passed through a sterile muslin gauze and washed through with PBS. Cell suspensions from both the trypsin and collagenase digests were pelleted, resuspended in PBS and combined. Cells were layered onto a Pancoll gradient (PAN-biotech) and spun for 20 minutes without brake at 3,000 rotations per minute (rpm). The leukocyte layer was collected and washed in PBS. Yolk sacs were washed in PBS, mechanically dissociated with scissors and digested in collagenase for 5 minutes, washed and filtered.<br><br>Flow cytometry and data analysis<br>Cell suspensions were stained for viability with 1:1000 LIVE/DEAD Fixable Blue (Thermo Fisher Scientific), or 1:1000 Zombie Aqua (Biolegend), both for 20 minutes at 4oC, and washed twice in PBS. For cell sorting cell suspensions were stained for viability with 1:3000 4',6-diamidino-2-phenylindole (DAPI) (Sigma-Aldrich) immediately before sorting.<br>Cells were blocked in human blocking buffer (5% human serum (Sigma-Aldrich), 1% rat serum (Sigma-Aldrich), 1% mouse serum (Sigma-Aldrich), 5% FBS and 2mM EDTA) for 15 minutes at 4oC and were incubated with antibody cocktails for 30 minutes at 4oC. Antibodies used are listed above and in Supplementary Table 1. Cells were washed and resuspended in FACS buffer (PBS containing 2% FBS and 2mM EDTA). The lineage (lin) channel in flow cytometry analyses included combinations of the markers CD3, CD19, CD20, CD41, CD56, CD66b, CD235a and CD335, for the removal of contaminating T cells, B cells, NK cells erythrocytes, megakaryocytes/platelets and granulocytes. Flow cytometry was performed using a Cytek Aurora (Cytek) or an Attune NxT (Thermo Fisher Scientific), or cells were purified by cell-sorting using a BD FACS Aria III (BD bioscience). Flow cytometry data was analysed using FlowJo v10.7 (Treestar), R version3.6.3 (The R foundation) and Prism 9 (GraphPad).<br>A gating strategy for the isolation of PEMP, HBC and any intermediates was identified using the Hyperfinder plugin in Flowjo, using default parameters. Down-sampled gated cells were exported from Flowjo, imported into R and transformed using the 'autoLgcl' transformation using the 'cytof_exprsMerge' function from the Cytofkit2 R package. The datasets were batch corrected using the 'mnnCorrect' function from the batchelor R package and subjected to PCA analysis using the 'prcomp' function.<br>Index-sort plate data was loaded into R using the 'retrieve_index' function from the indexSort R package, and the resultant data matrices were transformed via the 'logicleTransform' function in the flowCore R package (parameters; w = 0.6, m =4.2, a = 0). Cells which generated colonies were manually selected and transformed marker expression data was extracted for visualisation and statistical analysis in Prism 9 (GraphPad).
Instrument	Cytek Aurora (Cytek), Attune NxT (Thermo Fisher Scientific), BD FACS Aria III (BD bioscience)
Software	FlowJo v10.7 (Treestar), R version3.6.3 (The R foundation) and Prism 9 (GraphPad). Hyperfinder plugin in Flowjo
Cell population abundance	The abundance of PEMP decreases across gestation, from ~ 0.05% at 4 PCW to virtually absent by 9 PCW, as demonstrated in Figure 1F,G.<br>Post sort purity checks were performed placental macrophage and monocyte populations used for ATAQseq. Post sort purity checks were performed by acquiring ~100 events from sorted populations on the same FACS ARIAIII from which the samples were collected.<br>PEMP and GMP are too rare to carry out post-sort purity checks.

Gating strategy

PEMP were defined as DAPI-HLA-DR-FOLR2-CD41-Lin-CD66b-CD14-CD235a-CD34+CD43+ (Figure 1a)
GMP were defined as DAPI-Lin-CD34+CD38+CD10-CD45RA+ (Figure S6b)
First trimester HBC were defined as DAPI-CD45+CD14+Lin-HLA-DR-FOLR2+ (Figure S7b)
Term HBC were defined as DAPI-CD45+CD14+Lin-HLA-DR+/-FOLR2+ (Figure S9a)
Fetal blood monocytes isolated from placentall digests were defined asDAPI-CD45+CD14+Lin-HLA-DR+FOLR2- (Figure S9a)
PAMM1 were defined as DAPI-CD45+CD14+Lin-HLA-DR+FOLR2-CD9+CCR2- (Figure S7a and S9a)
Yolk sac macrophages were defined as DAPI-CD45+CD14+Lin-HLA-DR-FOLR2+ (Figure S7b)
Fetal were seperated from maternal cells using anti-HLA typing antibodies

☒ Tick this box to confirm that a figure exemplifying the gating strategy is provided in the Supplementary Information.
